# The distinctive psychopathology of NMDAR-antibody encephalitis compared with primary psychoses: an international, multicentre, retrospective phenotypic analysis

**DOI:** 10.1016/S2215-0366(25)00305-0

**Published:** 2026-01

**Authors:** Adam Al-Diwani, Jakob Theorell, Tarek Zghoul, Aniruddha Voruganti, Leigh Townsend, Riccardo De Giorgi, Benjamin Griffin, Tomasz Bajorek, David Okai, Belinda Lennox, M Isabel Leite, Carla Y Kim, Arielle Coughlin, Kelsey Martin, Brittany Glassberg, Christian Lachner, Nicola Westerbeek, Veerle Bergink, Kiran T Thakur, Anusha K Yeshokumar, Harald Prüss, Gregory S Day, Carsten Finke, Adam E Handel, Sanjay G Manohar, Dan W Joyce, Sarosh R Irani

**Affiliations:** a University Department of Psychiatry, University of Oxford, Oxford, UK; b Department of Experimental Psychology, University of Oxford, Oxford, UK; c National Institute for Health Research (NIHR) Oxford Health Biomedical Research Centre, Oxford Health NHS Foundation Trust, Oxford, UK; d Center for Infectious Medicine, Department of Medicine Huddinge, Karolinska Institutet, Stockholm, Sweden; e Department of Neurology, Karolinska University Hospital, Stockholm, Sweden; f Oxford Autoimmune Neurology Group, Nuffield Department of Clinical Neurosciences, University of Oxford, Oxford, UK; g Translational and Clinical Research Institute, Newcastle University, Newcastle-upon-Tyne, UK; h Brighton & Sussex Medical School, Falmer, UK; i Sussex Partnership NHS Foundation Trust, Brighton, UK; j Department of Psychological Medicine, John Radcliffe Hospital, Oxford University Hospitals NHS Foundation Trust, Oxford, UK; k Department of Neurology, John Radcliffe Hospital, Oxford University Hospitals NHS Foundation Trust, Oxford, UK; l Department of Neuropsychiatry, South London and Maudsley NHS Foundation Trust, London, UK; m Department of Neurology, Columbia University Irving Medical Center–New York Presbyterian Hospital, New York, NY, USA; n Department of Neurology, Icahn School of Medicine at Mount Sinai, New York, NY, USA; o Department of Psychiatry, Icahn School of Medicine at Mount Sinai, New York, NY, USA; p Department of Neurology, Mayo Clinic, Jacksonville, FL, USA; q Department of Neuroscience, Mayo Clinic, Jacksonville, FL, USA; r Department of Psychiatry, Erasmus Medical Center Rotterdam, Rotterdam, Netherlands; s Department of Neurology and Experimental Neurology, Charité, Universitätsmedisin Berlin, Berlin, Germany; t German Center for Neurodegenerative Diseases (DZNE) Berlin, Berlin, Germany; u Berlin School of Mind and Brain, Humboldt-Universität zu Berlin, Berlin, Germany; v Department of Primary Care and Mental Health, University of Liverpool, Liverpool, UK; w Mental health Research for Innovation Centre (M-RIC), University of Liverpool, and Mersey Care NHS Foundation Trust, Liverpool, UK

## Abstract

**Background:**

N-methyl-D-aspartate receptor (NMDAR)-antibody encephalitis is a life-threatening neuropsychiatric disorder requiring prompt immunotherapy. The earliest features are mental-state changes, often mistaken for primary psychosis. Improved clinical differentiation could assist rational diagnostic investigation and expedite immunotherapy. Inspired by patients’ and relatives’ lived experience, we aimed to explore the psychiatric phenotype of NMDAR-antibody encephalitis and the features common to and distinct from real-world episodes of psychosis.

**Methods:**

In this international, multicentre, retrospective phenotypic analysis we collected data on episodes of NMDAR-antibody encephalitis from specialised neurology services in Europe (UK, Germany, and Sweden) and the USA. For comparison, we collected similar data from de-identified accepted referrals to a UK early intervention in psychosis service, including consecutively presenting cases (unselected psychosis) and a group defined by having been assessed and admitted to hospital under the Mental Health Act (selected psychosis). Additionally, we included episodes of postpartum psychosis from a mother and baby unit in the Netherlands. In our mental-state inventory we included core features from ICD-11, the Bush–Francis catatonia score, and the Neuropsychiatric Inventory encompassing anxiety, depression, mania, schizophrenia, catatonia, and also more granular transdiagnostic behavioural features including those common to neuropsychiatric and neurobehavioural syndromes, such as delirium and dementia. Ethnicity data were not available. We compared and visualised the neuropsychiatric phenotype of these cohorts.

**Findings:**

We collected data from 100 episodes of NMDAR-antibody encephalitis from 96 patients between 2010 and 2022 (median age 22 years, female:male ratio 3·80), 135 episodes of psychosis from 135 patients between 2018 and 2019 (median age 27 years, female:male ratio 0·75), and ten episodes of postpartum psychosis from ten patients between 2005 and 2012 (median age 30 years, all female sex). Psychopathology in NMDAR-antibody encephalitis was abundant (92 [92%] of 100 episodes) and ultra-rapid in onset (median 1 day [95% CI 1–1; IQR 1–7]) versus unselected primary psychoses (median 180 days [120–210; 91–365]; p<0·0001). 21 (36%) of 58 mental-state features, including catatonic and visual hallucinations, were over-represented in NMDAR-antibody encephalitis and 12 (21%) were under-represented, including features typical of affective (eg, elated mood, flight of ideas, grandiose delusions) and non-affective psychoses (eg, thought broadcasting, thought withdrawal, paranoid delusions; false discovery rate threshold <0·05). Typically, in NMDAR-antibody encephalitis, the complexity sequentially evolved from mood to psychotic to catatonic predominance within 2 weeks.

**Interpretation:**

NMDAR-antibody encephalitis has a rapid-onset, complex, and dynamic neuropsychiatric phenotype, sufficiently distinctive to drive a clinical approach to differentiation.

**Funding:**

UK NIHR, Wellcome, and UK Medical Research Council (MRC)/UK Research and Innovation.

## Introduction

N-methyl-D-aspartate receptor (NMDAR) antibody encephalitis is a life-threatening neuropsychiatric disorder that most commonly affects young adults and is regarded as an important differential diagnosis of primary psychosis.^1^, ^2^, ^3^, ^4^ NMDAR-antibody encephalitis confounds the psychiatry–neurology dichotomy, traversing features encountered in patients with severe mental illness and in those managed by neurologists, with seizures, movement disorders, and autonomic dysfunction. The psychiatric disturbance is usually a heralding early feature, providing a window of diagnostic opportunity. Yet despite increasing awareness of NMDAR-antibody encephalitis globally, there remains considerable scope to leverage rational precision approaches to expedite definitive medical treatments, including immunotherapy and, when relevant, ovarian teratoma resection.^4^, ^5^

**Research in context**

**Evidence before this study**

The prompt and accurate differentiation of N-methyl-D-aspartate receptor (NMDAR)-antibody encephalitis from common primary psychoses remains a clinical problem. Diagnostic uncertainty can delay definitive immune treatment but also risk excessive potentially harmful over-investigation and unnecessary treatments. Before this study, based on our prior knowledge and a search of PubMed on Sept 22, 2025, using the search terms ((anti-N-methyl-d-aspartate OR NMDA receptor OR NMDAR OR NMDAR-antibody OR anti-NMDA OR anti-NMDAR OR NMDA OR NMDA-antibody) AND (encephalitis OR autoimmune encephalitis) for articles in published English, we were aware of systematic literature reviews and mostly single-centre studies describing the psychiatric expression of acute NMDAR-antibody encephalitis or neuroimmune findings in psychosis. There has been increasing published evidence that, consistent with our clinical experience, NMDAR-antibody encephalitis typically presents with psychiatric features, is rare in psychiatric settings, and its psychopathology might be atypical. There has been direct phenotypic comparison between the convalescent phase of NMDAR-antibody encephalitis and schizophrenia, but fewer comparisons for the early presenting phase in which diagnostic accuracy is paramount and predominantly relies on clinical observations. A consensus approach specifically for the diagnosis of NMDAR-antibody encephalitis requires access to physical investigations, accessible to the neurohospitalist, yet diagnostic delays usually occur before this stage. A concept derived from this consensus approach, autoimmune psychosis, aims to help identify NMDAR-antibody encephalitis and potential other currently undescribed autoimmune-mediated causes of psychosis, with, at the initial possible step, a purely clinical approach. The literature also contains red flag-type approaches and aide memoires from clinical experience, with some quantitative analyses describing the performance of these heuristics across comparator groups.

**Added value of this study**

We report the detailed psychopathology of NMDAR-antibody encephalitis from a substantial and well characterised international multicentre cohort through a bespoke inventory informed by both lived experience and traditional psychiatric features. By comparison with a real-world psychosis service, we show multidimensional features that bolster the argument for psychiatric atypicality at disease onset in NMDAR-antibody encephalitis. Our timeseries analysis of NMDAR-antibody encephalitis supports the increasingly recognised rapidity and complexity as characteristic disease hallmarks and highlights a temporal evolution of psychiatric features. We quantify the decision-making accuracy of heuristics for identifying autoimmune psychosis and show that these criteria, as well as simple, routinely noted psychopathological details, can help guide diagnostic suspicion in the acute phase.

**Implications of all the available evidence**

Our clinical observations, combined with studies reporting early cognitive changes in NMDAR-antibody encephalitis, suggest that psychosis concepts emphasising atypicality are most apt. Delirium, a neuropsychiatric syndrome encompassing fluctuant and rapid-onset cognitive and mental-state changes, often considered in older people with systemic disturbances, is probably an under-considered phenotypic model of the NMDAR-antibody encephalitis neurobehavioural syndrome. By analogy to Kraepelin's *dementia praecox*, we term this young-onset delirium as *delirium praecox*, which should be readily identifiable by its hyperacute, dynamic, and stereotyped accumulation of psychopathological features. Nonetheless, the rarity of NMDAR-antibody encephalitis in non-specialist settings compared with primary psychosis, particularly before its clinical evolution, makes rule-in certainty very challenging without objective laboratory tests. In most patients, NMDAR-antibody encephalitis manifests emergently, often with neurocritical features, and recognition at this overt level remains paramount, mandating urgent acute medical assessment. However, in patients for whom such overt neurological features are yet to occur or are less obvious, our findings support a clinically led approach, increasing access to cerebrospinal fluid analysis within psychiatric services. Future collaborative prospective designs should aim to quantitatively evaluate clinical and focused investigations that help advance simple scalable approaches to enhance the timely identification of NMDAR-antibody encephalitis and other potentially immunotherapy-modifiable mechanisms in psychotic disorders.

However, the task is challenging. First-episode psychosis also has a young adult onset and presents approximately 1500 times more commonly than NMDAR-antibody encephalitis (estimated incidence 45·00/100 000 person-years *vs* 0·03/100 000 person-years).^6^, ^7^, ^8^ First-episode psychosis generally has a multifactorial biopsychosocial aetiology wherein extensive laboratory investigation can be low in yield, risking overinterpretation of incidental findings.^9^ In the community settings where first-episode psychosis typically first emerges, the rarity of NMDAR-antibody encephalitis renders pre-test probabilities so low that, without considering clinical parameters, a screening laboratory test would need near-perfect discriminative accuracy to drive a likelihood ratio sufficient to overcome this very low prior probability of NMDAR-antibody encephalitis. To further complicate matters, commercially available serum testing of NMDAR autoantibodies carries a false-negative rate of up to 20%, making blood screening alone unsuitable.^10^ Although cerebrospinal fluid (CSF) testing is far more reliable, lumbar puncture remains challenging to deliver in many psychiatric centres.^10^, ^11^, ^12^ These factors underline the need to refine prior probability through clinically based case identification.^13^, ^14^, ^15^

The literature has increasingly suggested that the psychopathology of NMDAR-antibody encephalitis is atypical for primary psychosis.^16^, ^17^, ^18^, ^19^, ^20^, ^21^, ^22^ Thus, definition of a clinical signature is likely to have diagnostic usefulness. Here, inspired by patient narratives and our own clinical observations, we sought to test routine mental-state examination features to refine the differential diagnosis of NMDAR-antibody encephalitis. Aiming to extend a precision medicine approach to the diagnosis of NMDAR-antibody encephalitis in the acute setting, in an international collaboration we collected a multicentre NMDAR-antibody encephalitis cohort and, using de-identified electronic health-care records from psychosis episodes encountered in routine psychiatric clinical practice, we compared the clinical phenotype as directly as possible.

## Methods

### Study design and patients

The study was inspired by the vivid lived experiences reported to our clinical research team from participants and their carers, as well as experienced psychiatrists referring patients with suspected NMDAR-antibody encephalitis (appendix p 13).^23^

We conducted a multicentre, international, retrospective phenotypic analysis of the psychiatric features of the onset of NMDAR-antibody encephalitis in comparison with operationally defined primary psychoses. We compared the phenotype in episodes of NMDAR-antibody encephalitis from specialised neurology services across five regions in Europe (Oxford, UK; Berlin, Germany; Stockholm, Sweden) and the USA (New York, NY; St Louis, MO) versus episodes of primary psychoses from an early intervention in psychosis service (Oxfordshire and Buckinghamshire, UK) and a mother and baby unit (Rotterdam, Netherlands).

For NMDAR-antibody encephalitis and postpartum psychosis, patients gave written informed consent and were recruited according to approved local study ethical approvals (Oxford Research Ethics Committee 16/YH/0013 to SRI; Berlin EA1/258/18 to CF and HP; New York Institutional Review Board [IRB] AAAR3440 to KTT; 18-1744-00 to AKY; Washington University in St Louis IRB 201512120 to GSD; Stockholm Regionala etikprövningsnämnden i Stockholm Dnr 2009/2107-31/2 to JT; and Rotterdam NL58913.078.16 to VB). For the early intervention in psychosis service, after approval from the Oxford Health NHS Foundation Trust CRIS Oversight Committee (reference NMDAR, approved August, 2020), de-identified electronic health-care records (ie, clinical notes and, if applicable, correspondence including admission and discharge summaries) were retrieved and reviewed with the Oxford Health NHS Foundation Trust CRIS dataset.

We aimed to include patients of all ages who had episodes of definite NMDAR-antibody encephalitis, recruited into our research studies with sufficient data for phenotypic study to be ascertained as per consensus criteria.^24^ We retained patients who met probable criteria of NMDAR-antibody encephalitis based upon a typically presenting syndrome and supportive investigations including serum NMDAR-IgG, but for whom evidence of acute CSF NMDAR-IgG was unavailable.

To maximise face and ecological validity of the comparator episodes, we included patients with psychosis episodes based upon acceptance to the caseload of an early intervention in psychosis service. We sought to gather a sample sufficient in size and clinical properties to compare with our NMDAR-antibody encephalitis research database and thus extracted a sample of overall similar size within time and funding available. We used the Clinical Record Interactive Search (CRIS) to retrieve de-identified case records for referrals accepted by the Oxfordshire and Buckinghamshire Early Intervention Service (UK). We extracted consecutive episodes, to which we refer as unselected psychosis. To identify an enriched rapid-onset and severity subgroup so as to provide face overlap with NMDAR-antibody encephalitis, we identified a subgroup in whom a Mental Health Act assessment (ie, the legal framework regarding involuntary statutory admission to hospital in England and Wales) had been done within a month of acceptance to the service, resulting in compulsory psychiatric admission. Of the patients included in this subgroup, we extracted the first patients available who did not overlap with those already extracted, and refer to their episodes as selected psychosis. We sought to gather a sample sufficient in size and clinical properties to compare with our NMDAR-antibody encephalitis research database, thus we extracted a sample of overall similar size within time and funding available. Consistent with our approach and constraints of real-world data, we were not able to systematically rule out NMDAR-autoantibodies in these groups. Instead, we pragmatically accepted the very low incident rate of NMDAR-antibody encephalitis established previously^15^, ^25^ and noted the same investigation findings as our NMDAR-antibody encephalitis research database, if done (NMDAR-autoantibody testing in serum or CSF, electroencephalogram [EEG], MRI brain, and CSF protein and cell count). For postpartum psychosis, included on the basis of established rapidity and atypicality, we included inpatients with a new psychotic episode (affective or non-affective) in the postpartum period from the mother and baby inpatient unit of the Erasmus Medical Centre (Rotterdam, Netherlands). Given that some patients with postpartum psychosis have been found to have NMDAR-autoantibodies^26^ and that these data were available, we excluded patients with known serum NMDAR-autoantibodies.

### Measures

We developed a case report form for each illness episode covering demographic (ie, age and biological sex) and clinical details. The responding collaborative centre clinician investigators noted the presence and timing of established core clinical features, initial working diagnosis, investigations, and treatments (appendix p 1), followed by a study-specific psychopathology inventory (appendix pp 3–4). The minimum level of data for inclusion was inspection of health-care records covering the illness episode. We noted overt core clinical features of NMDAR-antibody encephalitis as per foundational case series and consensus criteria.^1^, ^2^, ^3^, ^24^ The timing of onset of these core features, including psychiatric, was considered to be the time taken for the feature to reach a level at which it was clearly present and of clinical severity (ie, causing distress to self and/or others). Together with these core clinical features, our psychopathology inventory was designed to test our previous literature-derived observation that NMDAR-antibody encephalitis was diagnosed with a range of primary psychotic and mood disorders, yet is a poor fit for them.^16^ To allow the identification of both typical and atypical patterns, we included core features from ICD-11, the Bush–Francis catatonia score, and the Neuropsychiatric Inventory encompassing anxiety, depression, mania, schizophrenia, catatonia, and also more granular transdiagnostic behavioural features including those common to neuropsychiatric and neurobehavioural syndromes, such as delirium and dementia. We coded these data as present, absent, or having no data (+1, –1, and 0), and we reported positive instances. For NMDAR-antibody encephalitis, we also collected data regarding presentation to clinical services and medications (appendix p 2).

### Statistical analysis

Data were tabulated in Microsoft Excel then cleaned and analysed in R (version 4.3), MATLAB (MathWorks, version 2024), and Prism (GraphPad, version 10.4). Analyses were conducted with available data (for completeness see appendix p 2). All comparisons were two-tailed. 95% CIs of medians were estimated by the binomial distribution method as implemented in Prism. We compared ages and timings of psychiatric onset between groups using a Mann–Whitney test (unpaired non-parametric column data) and proportions of past psychiatric or substance use disorder using Fisher's exact test. For mental-state examination features, we compared NMDAR-antibody encephalitis with pooled selected and unselected episodes from the early intervention service using Fisher's exact test and corrected for multiple comparisons with the Benjamini–Hochberg procedure (false discovery rate 0·05, a balanced conservative threshold consistent with the scope of our dataset and exploratory analysis).

For extended methods, see the appendix (pp 24–26). Briefly, to visualise how the pattern of features distinguished between individuals, we used an unsupervised dimensionality reduction method, *t*-distributed stochastic neighbour embedding (tSNE; MATLAB Stats toolbox). To analyse the evolution of NMDAR-antibody encephalitis over time, we plotted median onset timings of mental-state features with more than five available timings. To complement this analysis, we classified closeness of fit to a range of psychiatric disorder concepts to which NMDAR-antibody encephalitis has been compared using a non-competitive distance-normalised weightings approach and a two-dimensional reduction (mood *vs* others) to show trajectory through symptom space. To analyse and visualise mental-state feature co-occurrence, we made a co-occurrence matrix from which we generated chord diagrams with the *circlize* R package (thresholded at 30% of the group size for between-group visualisations and 20% together with feature selection by time of onset of up to 7 days and up to 20 days for the NMDAR-antibody encephalitis within-group visualisation).

To assess the utility of possible autoimmune psychosis as a diagnosis, a clinical-only stage of the decision rule that aims to help select patients with psychosis for physical investigation including CSF testing,^27^ we operationalised these criteria to align with our dataset. To estimate discriminative performance of clinical approaches in different settings, we created two comparator groups to simulate: (1) the caseload of a community early intervention team (unselected psychosis only); and (2) a pooled cohort with greater clinical diversity, including selected and postpartum psychoses plus unselected psychosis, to further challenge the classification rules. We used a two-step process of firstly having a psychiatric onset of less than 3 months and secondly having one or more clinical features in the rule (appendix p 10). Given that the approach has a time factor, we included episodes with available psychiatric onset timing. For episodes that were misclassified, we plotted the frequency of rule features that led to misclassification. In an orthogonal approach, we used a Bayesian general linear model to estimate the size and direction of these parameters and the initial step of possible autoimmune encephalitis (ie, subacute onset <3 months from major cognitive or altered mental status, interpreted here as a reduction in consciousness).^24^ To establish whether the mental-state examination data alone could discriminate as well as the possible autoimmune psychosis rule, we divided our dataset into training and test sets of equal size and composition and used a random forest-based tool to automatically discover features with the greatest value in differentiating between datasets. Finally, to consider the interaction between these approaches and the varied clinical contexts in which NMDAR-antibody encephalitis might be considered, we plotted positive and negative predictive values from 0% to 100% prevalence based upon 50% sensitivity and specificity, and those derived from the previous accuracy calculations.

### Role of the funding source

The funders of the study had no role in study design, data collection, data analysis, data interpretation, or writing of the report.

## Results

The initial search of CRIS retrieved 780 episodes of unselected psychosis occurring between January, 2018, and December, 2019, of which we extracted 83 consecutive episodes from 83 patients. The referrals for which an England and Wales Mental Health Act assessment was conducted and that resulted in psychiatric admission yielded 111 patients with selected psychosis occurring within 2018–19, of whom we extracted the first 52 patients available (52 episodes), selected to be a mimic of presentations of NMDAR-antibody encephalitis that did not overlap with those already extracted. Ten inpatients with postpartum psychotic episodes (ten episodes) within 4 weeks of delivery occurring between 2005 and 2012 were included from the Erasmus Medical Centre (Rotterdam, Netherlands; table).

**Table:** Cohort characteristics

		**NMDAR-antibody encephalitis**	**Unselected psychosis**	**Selected psychosis**	**Postpartum psychosis**
Number of illness episodes		100	83	52	10
Number of patients		96	83	52	10
Episodes per reporting centre; number of patients*					
	Oxford, UK	12; 11	83; 83	52; 52	NA
	Berlin, Germany	45; 45	NA	NA	NA
	New York, NY, USA	29; 28	NA	NA	NA
	St Louis, MO, USA	9; 9	NA	NA	NA
	Stockholm, Sweden	5; 3	NA	NA	NA
	Rotterdam, Netherlands	NA	NA	NA	10; 10
Median age of episode onset, years (range)		22 (2–68)	26 (15–58)	29 (13–52)	30 (21–41)
Sex					
	Female	76/96 (79%)	33/83 (40%)	25/52 (48%)	10/10 (100%)
	Male	20/96 (21%)	50/83 (60%)	27/52 (52%)	0
Inpatient admission per episode					
	Psychiatric hospital	36/100 (36%)	17/83 (20%)	52/52 (100%)	10/10 (100%)
	General hospital	100/100 (100%)	0	0	0
Positive NMDAR-IgG test per episode tested					
	Serum	61/77	1/11†	1/13‡	0/10
	CSF	98/100	NA	0/1	NA
NMDAR-antibody encephalitis-specific illness associations					
	Patients with ovarian teratoma	25/96 (26%)	NA	NA	NA
	Patients post-HSV encephalitis	2/96 (2%)	NA	NA	NA

Data are n, n/N (%), or median (range). CBA=cell-based assay. CSF=cerebrospinal fluid. HSV=herpes simplex virus. NA=not applicable. NMDAR=N-methyl-D-aspartate receptor.

* Seven NMDAR-antibody encephalitis patients previously reported in Al-Diwani and colleagues (2019; n=5),^23^ Brier and colleagues (2016; n=1),^28^ and Kim and colleagues (2018; n=1).^29^

† Five research assays used live CBA (research); single positive result from fixed CBA (clinical) of non-encephalitic cause.

‡ Four research assays used live CBA; single positive on live (research) and fixed (clinical) CBA; CSF normal and negative for NMDAR-IgG, and electroencephalogram showed no encephalopathy.

**Figure 1:**
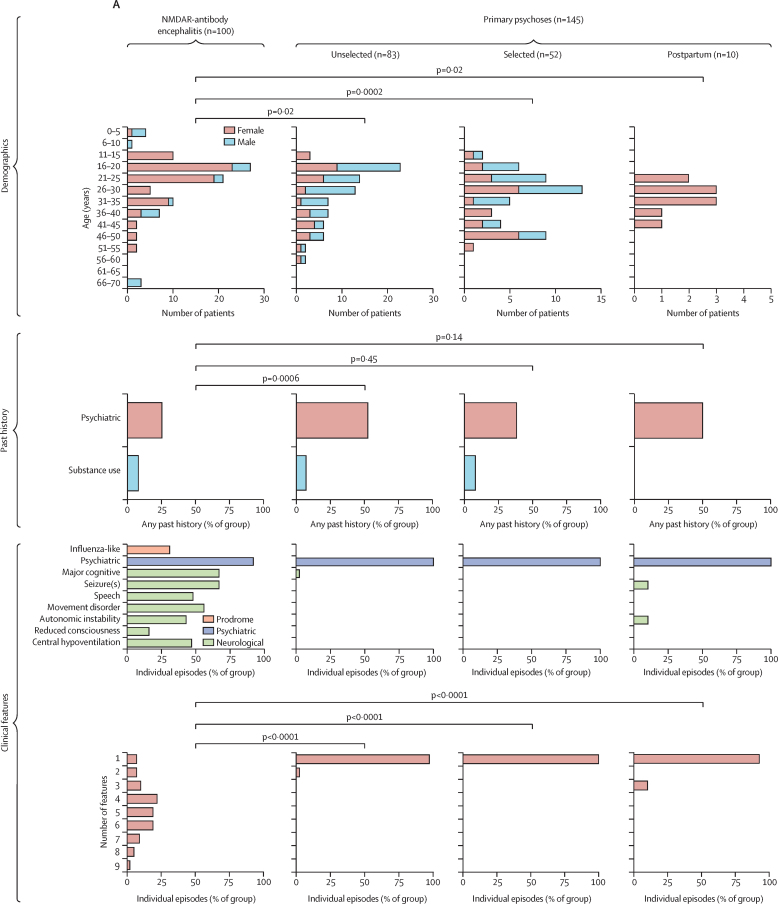

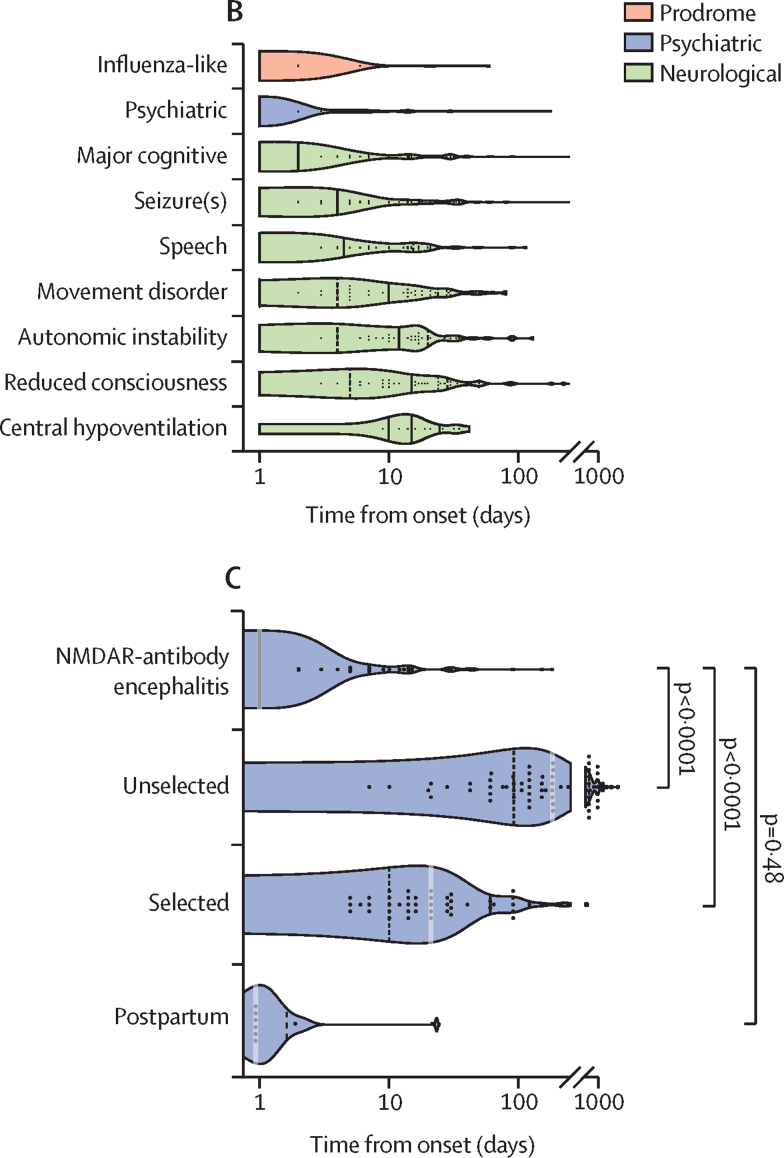
Clinical characteristics of NMDAR-antibody encephalitis and primary psychosis cohorts (A) Demographics, past psychiatric and substance use history, clinical feature count, and number of features per individual are plotted for NMDAR-antibody encephalitis versus three primary psychosis comparator groups. Age and features per individual are compared with a two-tailed Mann–Whitney test and past psychiatric history with a two-tailed Fisher's exact test. (B) The onset times of nine core NMDAR-antibody encephalitis clinical features are summarised by a violin plot on a logarithmic time axis (dots indicate syndrome onset time in individual illness episodes, and the vertical black bar indicates the median). The features are ranked from top to bottom by median time of onset. (C) The onset time of the NMDAR-antibody encephalitis psychiatric syndrome is compared with the primary psychosis groups (dots indicate syndrome onset time in individual illness episodes and the vertical white bar indicates the median). The median onset time of NMDAR-antibody encephalitis is compared with the comparator conditions with a two-tailed Mann–Whitney test. NMDAR=N-methyl-D-aspartate receptor.

We assessed 100 illness episodes of NMDAR-antibody encephalitis occurring between 2010 and 2022, from 96 patients across five regions in Europe and the USA. These episodes were compared with the 145 episodes of primary psychosis from 145 patients. We retained two patients who met probable criteria for NMDAR-antibody encephalitis for whom evidence of acute CSF NMDAR-IgG unavailable: one patient did not undergo CSF sampling and the other was tested after having fully recovered, with no detectable NMDAR-IgG in CSF, a reported finding.^30^ Our NMDAR-antibody encephalitis cohort was young (median age of onset 22 years [range 2–68]) and had more female patients (76 [79%] of 96) than male patients (20 [21%]; female:male ratio 3·80; median age of initial illness 22 years [95% CI 20–23], range 2–68; figure 1A; table). Young adults predominated in both unselected (median age 26 years [95% CI 23–29], female:male ratio 0·66) and selected psychosis (median age 29 years [26–32], female:male ratio 0·92) cohorts but without female predominance (figure 1A; table). A past history of any psychiatric condition was noted in 25 (26%) of 96 patients with NMDAR-antibody encephalitis (figure 1A) versus 43 (52%) of 83 in the unselected psychosis group (p=0·0006, Fisher's exact test) without statistically significant differences to the selected (17 [33%] of 52; p=0·45) and postpartum cohorts (five [50%] of ten; p=0·14). Overall, any previous psychiatric condition was less common in the NMDAR-antibody encephalitis group (odds ratio [OR] 0·43 [95% CI 0·25–0·75]; p=0·0042, two-sided Fisher's exact test). However, this difference provided only modest diagnostic accuracy (sensitivity 0·26, specificity 0·55, likelihood ratio 0·58). Unsurprisingly, the most common specific previous conditions across the cohorts were prevalent mental health conditions such as anxiety and depression, collectively accounting for 64 (63%) of 102 conditions (appendix p 5). In the NMDAR-antibody encephalitis group, six (21%) of the 29 previous psychiatric diagnoses were more severe forms of mental illness (schizophrenia n=1 and bipolar disorder n=5). Similar proportions endorsed a history of substance use difficulties in the NMDAR-antibody encephalitis and both early intervention in psychosis groups (NMDAR-antibody encephalitis: eight [8%] of 96; unselected psychosis: six [7%] of 83; selected psychosis: four [8%] of 52), and none were noted in the smaller postpartum psychosis group.

The commonest core clinical feature in the NMDAR-antibody encephalitis cohort was psychiatric, described in 92 (92%) of 100 illness episodes and, together with non-specific influenza-like symptoms (31 [31%]), it was the earliest feature (median onset of 1 day [95% CI 1–1; IQR 1–7]; figure 1A, B). Psychiatric features were either the only presenting feature or part of the presenting syndrome in 61 (61%) and were the next earliest feature in a further 23 (23%) of the illness episodes. In individuals with NMDAR-antibody encephalitis, up to nine core clinical features were identified, with a median of five per episode (95% CI 4–5, range 1–9; mode 4; figure 1A). Typically, these clinical features began just a few days after the onset of psychiatric features (figure 1B). In comparison, the primary psychoses were restricted to a singular psychiatric syndrome in almost all patients (median 1, mode 1; p<0·0001 for all via Mann–Whitney test; figure 1A). Only five (5%) of 100 NMDAR-antibody encephalitis episodes had a single core clinical feature: three were paediatric seizure disorders and two episodes were restricted to psychiatric features, which were relapses after a previous polysymptomatic presentation.

The absolute time of symptom onset was especially rapid in patients with NMDAR-antibody encephalitis and postpartum psychosis, with a median of 1 day in both (NMDAR-antibody encephalitis: 95% CI 1–1, IQR 1–7; postpartum psychosis: 1–22, 1–2), whereas unselected psychosis episodes were far slower in onset (median 180 days [95% CI 120–210; IQR 91–365]; p<0·0001 Mann–Whitney test) with only six (9%) of 67 episodes that had available onset times occurring in fewer than 30 days (figure 1C). Even episodes intentionally selected for rapid onset and severity showed largely distinct times to onset (median 21 days [95% CI 14–30, IQR 10–60]; p<0·0001 Mann–Whitney test). A cutoff of fewer than 7 days offered considerable value to differentiate NMDAR-antibody encephalitis from selected psychoses (OR 28·4 [95% CI 9·4–77·7]; p<0·0001; likelihood ratio 8·5) and, at 30 days, from unselected psychoses (82·4 [27·8–249·3]; p<0·0001; 11·3).

**Figure 2:**
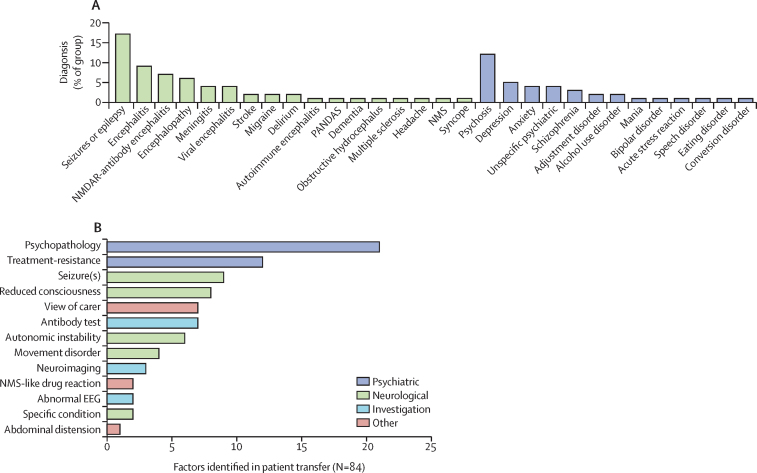
Real-world diagnostic processes in NMDAR-antibody encephalitis (A) Frequencies of initial working diagnoses in NMDAR-antibody encephalitis expressed as a percentage of available data are ranked from most to least frequent within broad neurological or psychiatric categories. These diagnoses are given as close to verbatim as possible, without reclassification, to represent real-world processes. (B) For patients with NMDAR-antibody encephalitis who were initially admitted to psychiatric care (n=36), the frequency of factors identified as relevant to transfer out are ranked from most to least frequent. EEG=electroencephalogram. NMDAR=N-methyl-D-aspartate receptor. NMS=neuroleptic malignant syndrome. PANDAS=paediatric autoimmune neuropsychiatric disorders associated with streptococcus infection.

In our analysis of patient journeys through clinical services, for many patients clinical recognition appeared sufficient to initiate empirical immunotherapies. For example, 31 (46%) of 67 patients in whom immunotherapy timing was available received steroids before a CSF sample was sent for antibody testing (appendix p 15). Nonetheless, in 78 (79%) of 99 available episodes, the initial working diagnosis differed from either NMDAR-antibody encephalitis or encephalitis or encephalopathy (figure 2A). In patients with NMDAR-antibody encephalitis, the initial impression in 32 (32%) of 99 episodes was of a psychiatric condition, including both psychosis and mood categories, and the most common neurological impression was seizures or epilepsy (17 [17%] of 99) rather than a specific pathology (figure 2A). When we specifically reviewed the 36 (38%) of 96 patients with NMDAR-antibody encephalitis initially admitted to a psychiatric ward, the reasons for transferring patients to a general medical setting were largely clinical, including mental-state features, treatment resistance, seizures, and reduced consciousness, in addition to the impression of carers (72 [86%] of 84 factors stated), rather than investigation results (12 [14%] of 84 factors stated; figure 2B). For most patients (25 [69%] of 36), more than one factor contributed to decision making (appendix p 17).

**Figure 3:**
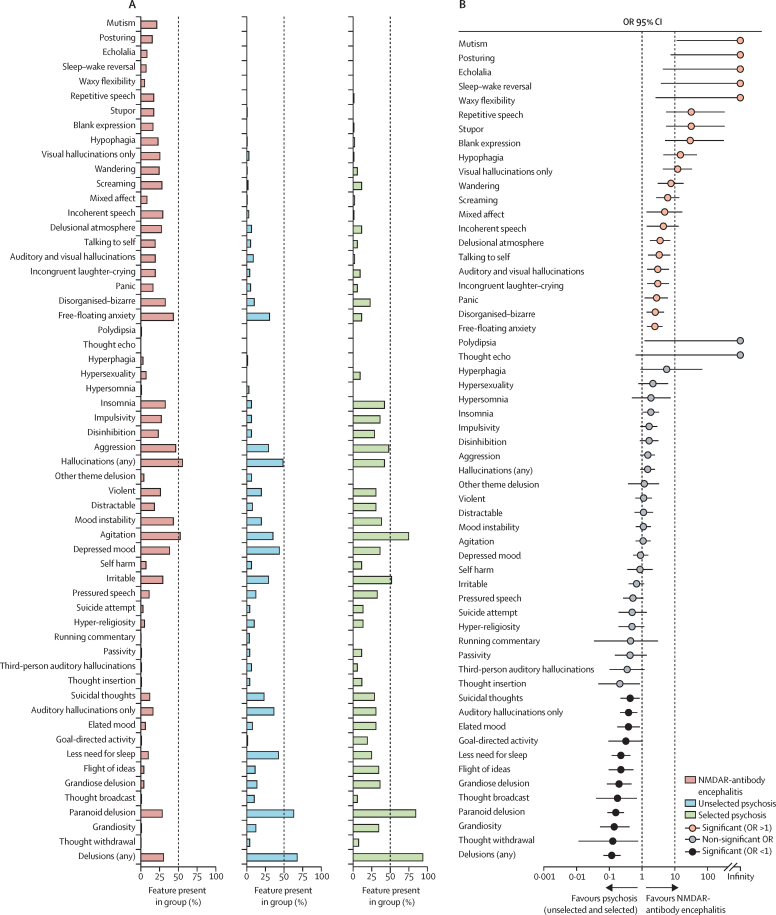

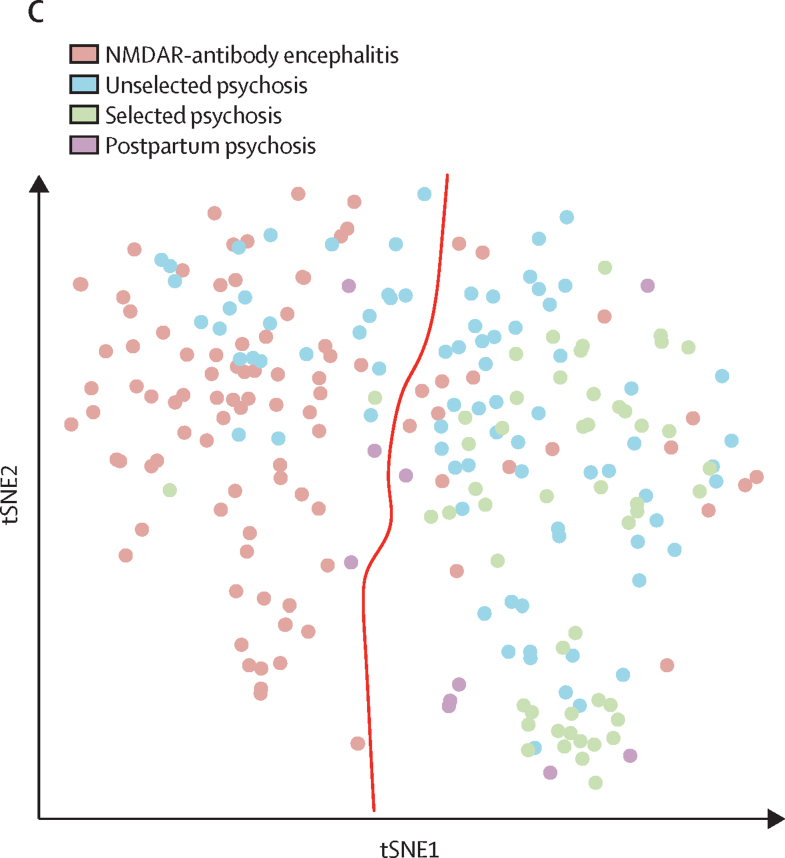
Mental-state features of NMDAR-antibody encephalitis versus early intervention in psychosis cohorts (A) The percentage of NMDAR-antibody encephalitis and unselected and selected psychosis groups expressing a series of mental-state features at any point in an illness episode. (B) Each mental-state feature proportion in NMDAR-antibody encephalitis is compared with the pooled unselected and selected psychosis groups. Univariate ORs are plotted with 95% CIs. Features significant after correction for multiple comparisons are shown in red (OR >1) and black (OR <1). ORs non-significant after correction are shown in grey. ORs with a denominator of 0 (ie, exclusively present in one group) are plotted as infinity. (C) tSNE plot in which the high-dimensional mental state of each illness episode is reduced to two dimensions and plotted relative to the others. The line divides the area into two populations in which >75% (left) or <75% (right) of the episodes are NMDAR-antibody encephalitis. Dots represent each episode. NMDAR=N-methyl-D-aspartate receptor. OR=odds ratio. tSNE=*t*-distributed stochastic neighbour embedding.

Using our bespoke mental-state inventory, we found that NMDAR-antibody encephalitis spanned all 58 features, of which many, but not all, could be found in the early intervention in psychosis cohorts: 49 (84%) in unselected psychosis and 46 (79%) in selected psychosis (figure 3A). Although the range of psychopathological features in NMDAR-antibody encephalitis was wide, for single features to predominate was rare, with only hallucinations and agitation present in 50% of episodes or more. In contrast, the range of features in primary psychoses was narrower and a few features were present in 50% of episodes or more: delusions (58 [70%] of 83 in unselected, 50 [96%] of 52 in selected) and paranoid delusions (54 [65%] in unselected, 45 [87%] in selected) in both groups; hallucinations (42 [51%]) in unselected psychosis; and aggression (26 [50%]), irritability (28 [54%]), and agitation (40 [77%]) in selected psychosis.

Comparing NMDAR-antibody encephalitis with pooled patients from the early intervention service, there were large ORs for several features favouring NMDAR-antibody encephalitis (figure 3B). The most stark relative differences (ie, OR >10) were largely catatonic features (motor and speech signs such as mutism, posturing, echolalia, and waxy flexibility, as well as sleep–wake reversal, which were exclusive to our NMDAR-antibody encephalitis cohort), with considerable enrichment for repetitive speech and stupor (both OR 31·8 [95% CI 5·4–334·7]; p<0·0001), blank expression (29·8 [5·0–314·0]; p<0·0001), and isolated visual hallucinations (12·3 [4·4–33·4]; p=0·0001; figure 3B). There were several features commonly observed in NMDAR-antibody encephalitis, including agitation, aggression, mood instability, pressured speech, hypersexuality, and insomnia, that were similarly common in primary psychosis after multiple comparison corrections (figure 3B; appendix pp 6–7). However, in the NMDAR-antibody encephalitis cohort, there were notably low frequencies of both first-rank symptoms of schizophrenia (eg, paranoid delusions, thought broadcast, thought withdrawal, and auditory hallucinations) and core features of mania (eg, flight of ideas, grandiose ideas, reduced sleep, and manic mood) that were far more common in the primary psychoses (figure 3B). In our analysis of feature co-occurrence, 15 features had more than 30% co-occurrences in NMDAR-antibody encephalitis, which reduced to 13 features in selected psychosis and six features in unselected psychosis (appendix p 18). Consistent with these contrasting pictures, dimensionality reduction identified more than 75% of NMDAR-antibody encephalitis episodes that clustered together and away from the primary psychoses episodes (figure 3C). Overall, although clearly showing some overlaps with primary psychoses, NMDAR-antibody encephalitis differed by an over-representation of mental-state features (21 [36%] of 58), including neurobehavioural features such as wandering and screaming and catatonic features such as mutism and posturing, and an under-representation of mental-state features (12 [21%]), including both an absolute and relative sparsity of core features observed in affective (eg, elated mood, flight of ideas, grandiose delusions) and non-affective psychoses (eg, thought broadcast, thought withdrawal, paranoid delusions).

**Figure 4:**
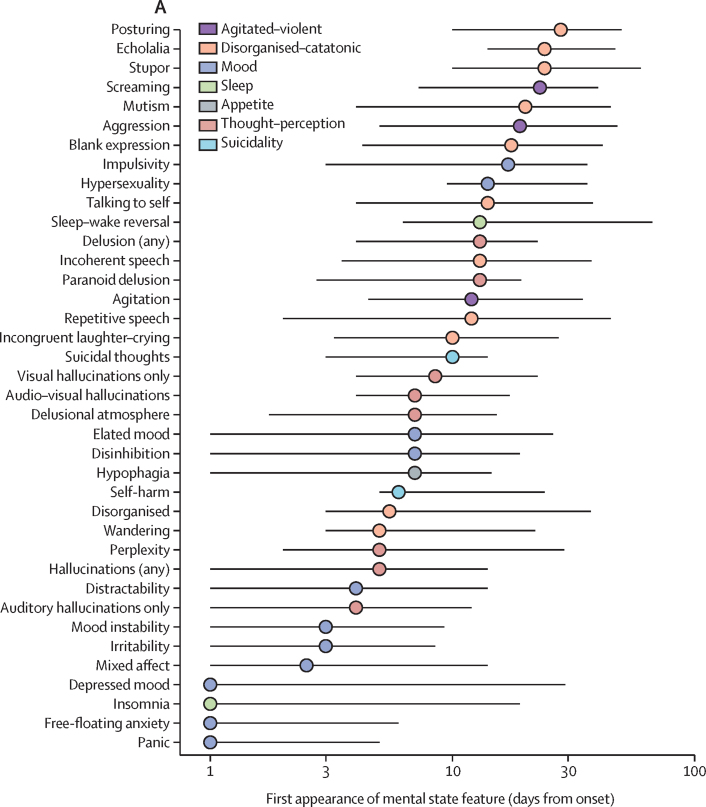

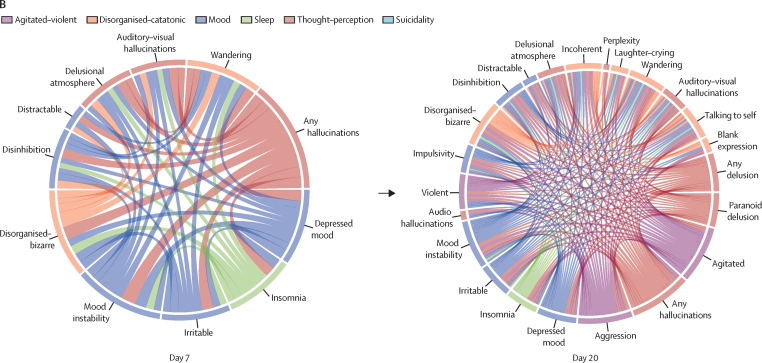
Mental-state features of NMDAR-antibody encephalitis over time (A) Median onset times (and IQR) of mental-state features with a threshold of more than five available episode times are plotted on a logarithmic time axis vertically ranked from earliest at the bottom to latest at the top. The colours refer to grouping the granular features into broader groups. (B) Within an episode, pairs of co-occurrent mental-state features are summarised in chord diagrams with a threshold of >20% cohort size cooccurrence. Each ribbon represents the occurrence of at least one pair. NMDAR=N-methyl-D-aspartate receptor.

For our analysis of the mental-state feature temporality in our NMDAR-antibody encephalitis cohort, 504 (46%) onsets were available for 1100 present features, of which 330 (65%) had offsets available. The earliest features—all with a median onset of 1 day relative to baseline—included panic, anxiety, depressed mood, and insomnia (figure 4A). The next few days were associated with the appearance of further mood features (ie, mixed affect, irritability, mood instability, and distractibility) and, psychotic features emerged shortly after (ie, hallucinations and perplexity), together with behavioural disturbance (ie, wandering and disorganisation). Thereafter, additional features from these groups were observed (ie, disinhibition, elation, delusional atmosphere, mixed auditory–visual hallucinations, and suicidality [ie, self-harm and suicidal thoughts]). In the remainder of the first month, further clusters emerged including agitated–violent (agitation, aggression, and screaming) and disorganised–catatonic behaviours (incongruent laughter–crying, repetitive and incoherent speech, talking to self, mutism, stupor, echolalia, and posturing).

When we evaluated these patterns in terms of the diagnoses to which NMDAR-antibody encephalitis has been compared (appendix pp 8–9, 19), closeness-of-fit analysis showed a temporal shift from mood disorders without psychosis, fitting best within the first 3 days, to mood disorders with psychotic features fitted better during week 1, in turn giving way to increasingly atypical psychoses by week 2. By week 3 onwards, disorganised–catatonic disorders had the highest model fit. Co-occurrence analyses, restricted to features of which the median presentation was by 7 days or 20 days, exemplified this shift with a marked increase in co-occurrence by day 20 (figure 4B). When we visualised this trajectory using a two-dimensional symptom space comprising a mood component plotted against a combined behaviour–thought–perception–speech component (appendix pp 19–20), changes within the first week were largely along a mood axis. Thereafter, these changes diminished, together with the emergence of other features. We found that the symptom space traversed in NMDAR-antibody encephalitis was even greater than in postpartum psychosis, despite being similarly abrupt in onset (appendix p 19).

**Figure 5:**
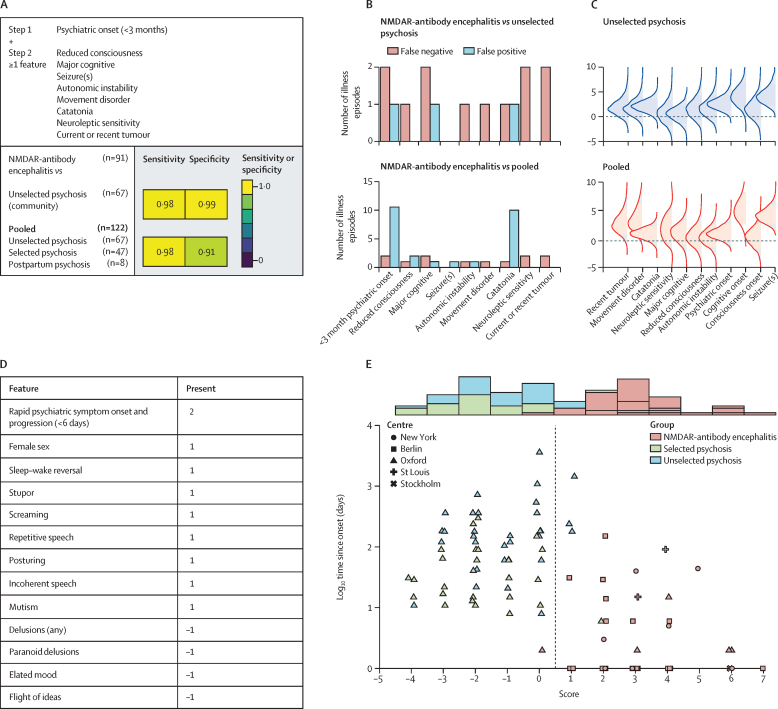
Performance of clinical decision rules to differentiate NMDAR-antibody encephalitis from primary psychoses (A) Sensitivity and specificity after the implementation of criteria for possible autoimmune psychosis to classify NMDAR-antibody encephalitis episodes with a psychiatric syndrome (n=91 with onset time available of 92 episodes with a psychiatric feature) versus unselected psychosis episodes only (ie, community; n=67) or pooled unselected, selected, and postpartum psychosis episodes (n=122). (B) For each classification exercise, features that were present driving incorrectly classified episodes are plotted as bar charts (associated with false-positive and false-negative outcomes). (C) Ridge plots of posterior distributions for each decision rule factor from possible autoimmune psychosis and initial clinical-only step of possible autoimmune encephalitis implemented for our dataset (ie, onset and progression of <3 months of cognitive, psychiatric, or reduced consciousness), modelled independently with shading indicating the 80% CI area. (D) Derived shortlist of features with score weighting. (E) Results when score is applied on the test subsets. The dashed line indicates the optimal threshold identified for the training set (ie, >1), offset slightly for illustrative purposes. The time since onset is given because this feature is the most discriminative, log_10_ transformed after addition of 1 to all values. Values are jittered to facilitate visualisation. Above the plot, a frequency distribution summarises score frequency and clinical group. Symbols indicate the centre from which the episode was derived. NMDAR=N-methyl-D-aspartate receptor.

We were able to compare primary psychosis episodes to NMDAR-antibody encephalitis episodes with psychiatric features at onset, with timing in most of our cohort (91 [91%] of 100; appendix p 2). The pooled cohort included 67 (81%) of 83 unselected psychosis episodes, 47 (90%) of 52 selected psychosis episodes, and eight (80%) of ten postpartum psychosis episodes that had available onset times (n=122). Consistent with the marked differences in phenotype, we found high sensitivity (0·98) for both comparisons and especially high specificity (0·99) for community (unselected psychosis) episodes, which was slightly lower in the pooled cohort (0·91; figure 5A). The decision rule features driving the false-positive events and reducing specificity were largely onset less than 3 months and catatonia (figure 5B). The episodes with NMDAR-antibody encephalitis classified as false negatives were atypical by having a psychiatric onset longer than 3 months. Consistent with this performance, the Bayesian generalised linear model found that all clinical factors had parameter estimates with widely dispersed posterior distributions that largely overlapped, pointing towards the need for combinatorial factors (figure 5C). Because our analysis of psychopathological patterns identified signals of apparent discriminative value in concert, rather than independently as in the generalised linear model, we asked if these phenomena alone could adequately differentiate between NMDAR-antibody encephalitis and primary psychoses as per our focus on earliest differentiation.

When we focused on mental-state features and divided our dataset into training and test sets to find features with the greatest differentiating value, we found that rapid onset of psychiatric features (<6 days), female sex, and 11 psychopathological features had the most discriminative value, showing stability between training and test sets (appendix p 21). Consistent with our initial descriptive analysis, the psychopathological features positively favouring NMDAR-antibody encephalitis were largely catatonia or delirium-like, reflecting psychomotor, speech, and circadian features (ie, mutism, incoherent and/or repetitive speech, posturing, screaming, stupor, and sleep–wake reversal), whereas those identifying primary psychosis episodes preferentially over NMDAR-antibody encephalitis were closely related to core features of severe mental illness (ie, elated mood, flight of ideas, any delusion, and paranoid delusions).

Using these observations, we derived a score most strongly weighted on ultra-rapid onset (2 points), with 1 point each for any of female sex or seven catatonic and neurobehavioural features over-represented in NMDAR-antibody encephalitis. 1 point is subtracted for each of any four core features of psychosis and mania that were over-represented in primary psychosis (figure 5D; appendix p 11). With the optimal cutoff score of less than 1 for the training data, this analysis produced a balanced accuracy of 97% (95% CI 0·92–0·99; sensitivity 0·98, specificity 0·96) in the training set and 95% (0·89–0·98; sensitivity 0·98, specificity 0·93) in the test set. There were no significant differences in score performance between the NMDAR-antibody encephalitis reporting centres (Mann–Whitney test: p>0·05 for all, range 0·21–0·84).

Finally, predictive values (appendix pp 12, 22–23) modelled on low and higher prevalence settings showed that these clinical approaches could bolster negative predictive values (negative predictive value ≥99% at a prevalence of 1% and 5%; ≥98% negative predictive value at a prevalence of 50%). Despite considerable improvements in positive predictive value with increasing prevalence, the absolute values were, as expected, less definitive. For example, at 1% prevalence, possible autoimmune psychosis tested against the community (unselected psychosis) cohort gave a positive predictive value of 50%, whereas the more conservative test derived from the comparison with the pooled psychosis yielded 10%, consistent with the similarly tested psychopathology approach (12%).

## Discussion

Our large, multicentre phenotypic analysis highlights the distinctive neuropsychiatric phenotype of NMDAR-antibody encephalitis directly and by comparison with primary psychoses. The complex and mixed psychopathology of NMDAR-antibody encephalitis shows an ultra-rapid (ie, <7 day) onset and dynamic progression from initial mood dominance through psychotic, disorganised, and catatonic phases, and, consistent with previous reports, occurs very rarely without overt neurological features.^31^, ^32^ Our findings help to explain why the psychopathology is challenging to classify with traditional constructs and why the condition is prone to diagnostic uncertainty, particularly in its early phase. Clinically based reductive approaches can help to manage this uncertainty and promote rational physical investigation and referral discussions. We anticipate our analysis, together with others, will prompt further conversations between patient groups, clinicians, and service managers to identify patients with an actionable, diagnostic previous probability while not overburdening patients with markedly low pre-test probabilities with invasive tests.

The ultra-rapid onset of the psychiatric component of NMDAR-antibody encephalitis is strikingly different to the psychosis episodes in our comparison group, with the exception of postpartum psychosis. The psychopathology that usually develop over months and years in most of the consecutively presenting unselected psychosis subgroup appears in only days and weeks in NMDAR-antibody encephalitis, a prime feature which contributes to the distinctiveness of the phenotype. This abrupt onset forms a key and simple part of the clinical approach that we advance, consistent with previous expert recommendations.^33^ However, this psychiatric opportunity is short and, in most patients, overt neurological features emerge after just a few days. This abruptness, combined with catatonia and neurological features, discriminated NMDAR-antibody encephalitis and most consecutively presenting cases of psychosis from early intervention cases, compatible with a previously proposed algorithm for psychiatric services.^15^ We found that features of the mental state represented the most common driver to consider the diagnosis for patients initially admitted to psychiatric care. In the absence of overt neurological features, the mental state can enrich diagnostic likelihood, which our heuristic approach could capture in an implementable form. However, if aiming for the earliest possible diagnosis, and if tools are applied without clinical judgement, a sizeable proportion of presentations could generate what transpire to be false positives after physical investigation. Overall, a decisive weight of abruptness and atypicality takes around 1–4 weeks to evolve and inherently requires some longitudinal observation. Thus, the field might be nearing a limit on empirical diagnosis to expedite the administration of immunotherapy. Given the pitfalls of screening with serum antibody testing and limitations in accessing corroborating tests such as EEGs, a timely rather than an early diagnosis might be the more realistic aim.

The key limitations of our study are the largely retrospective data collection and reliance upon electronic health-care records for the primary psychosis cases. First, for the NMDAR-antibody encephalitis group, to enable the benefits of size and diversity in multicentre descriptions and considering the relative rarity of NMDAR-antibody encephalitis, even at specialist centres, our retrospective observations were realistic and deliverable but might be less accurate than prospective studies. Second, we compared multicentre, multinational NMDAR-antibody encephalitis data with a single regional psychosis service in the UK; future studies should aim to have greater diversity in comparator groups and collect data on race and ethnicity. Third, our temporal analysis of granular features did not have start times for all features in all episodes, and stop times were sometimes unavailable. However, assuming features continue would be expected to underestimate rather than overestimate the dynamic quality of the syndrome. Fourth, our sample sizes are relatively modest in absolute terms. For our psychosis comparator group, we chose to favour a similar sample size to the NMDAR-antibody encephalitis group size that permitted data extraction by board-certified clinicians rather than generating greater numbers but relying upon language-processing software. Although improving rapidly, such software, in our judgement, is yet to reliably show this nuanced yet realistic extraction from psychiatric notes, which is paramount for a detailed and clinically translatable study of psychopathology.^34^ However, given that cohort and case–control studies are very challenging in this field due to the low population incidence of NMDAR-antibody encephalitis and the nature of the psychopathology complicating detailed prospective research-level study, computational approaches such as large language models might become more relevant soon. Finally, our study included cognitive dysfunction at an overt level of deficit, including profound disorientation, inattention, and anterograde amnesia, a level of deficit found in delirium and dementia disproportionate to the acknowledged cognitive aspects of primary psychoses. During our study analysis, a single-centre, prospective NMDAR-antibody encephalitis study showed that delirium criteria are usually satisfied and suggested that these criteria could help clinical recognition.^22^ We chose psychosis presenting to a specialist psychosis service as a comparator group because, due to similar age and syndrome, it represents a clinical service design without quick and easy access to neurological investigations, which could contribute to risk of delayed diagnosis. Although we did find psychopathological overlaps with primary psychosis, the clarity of differences we identified could support the idea that the frequent comparison with young-onset psychosis is a misframing, at least in part. Had we included a delirium group, it might have been harder to distinguish, encompassing both abruptness and overt cognitive features together with an under-appreciated range of psychopathology.^35^ Yet, delirium is uncommon in younger people outside of major physical illness and is not usually the first clinical consideration without overt symptoms of physical illness. Therefore, we suggest that framing this unusual atypicality as delirium of the young, or *delirium praecox*, could help to assist recognition.

Our findings renew emphasis on the role of clinical acumen as a test in its own right for this rare but severe and highly treatable autoimmune neurological disease. Although our data are detailed and quantitative, the reduction to an existing proposed clinical decision rule supports real-world rationalisation of physical investigation in people experiencing new-onset severe mental illness. Our score is simple to action and amenable to online implementation. The field now needs to test and evolve these approaches through collaborative multicentre prospective studies, aiming to progress clinical decision rules towards the timeliest instigation of disease-modifying treatment.

### Contributors

### Data sharing

De-identified data supporting the study are available from the corresponding author upon reasonable request after publication. CRIS source data for this work are owned by Oxford Health NHS Foundation Trust that uses anonymised patient records via CRIS powered by Akrivia Health. The data cannot be made publicly available, but can be accessed with permissions from Oxford Health NHS Foundation Trust for UK NHS staff and UK academics within a secure firewall, in the same manner as the authors.

## Declaration of interests

JT reports no competing interests directly relevant to this work, but has received honoraria from Lundbeck and SVAR laboratories for lectures on topics not related to the current manuscript. AKY is currently a full-time employee of Bristol Myers Squibb, but was not at the time this work was completed, and there is no overlap between her current work and that found in this manuscript. GSD reports no competing interests directly relevant to this work, but serves as a consultant for Arialys Therapeutics and Parabon Nanolabs and as a topic editor (dementia) for DynaMed (EBSCO); has developed educational materials for Continuing Education and Ionis Pharmaceuticals; owns stock in ANI pharmaceuticals; and has received in-kind contributions for radiotracer precursors for tau-PET neuroimaging in studies of memory and ageing from Avid Radiopharmaceuticals, paid to the institution. SRI has received honoraria and research support from UCB, Immunovant, MedImmun, Roche, Janssen, Cerebral therapeutics, ADC therapeutics, Brain, CSL Behring, and ONO Pharma; has licensed royalties on patent application WO/2010/046716; and has filed two other patents (WO2019211633 and US-2021-0071249-A1, Patent Cooperation Treaty application WO202189788A1; and PCT/GB2022/050614 and WO202189788A1). All other authors declare no competing interests.
