# Supplementary material for: The distinctive psychopathology of NMDAR-antibody encephalitis compared with primary psychoses: an international, multicentre, retrospective phenotypic analysis
Source: Lancet Psychiatry. 2026 Jan;13(1):47–61. doi: 10.1016/S2215-0366(25)00305-0 (PMC13003407; doi:10.1016/S2215-0366(25)00305-0)
Supplement: Supplementary appendix [file mmc1.pdf]

THE LANCET
Psychiatry

Supplementary appendix

This appendix formed part of the original submission and has been peer reviewed. We post it as supplied by the authors.

Supplement to: Al-Diwani A, Theorell J, Zghoul T, et al. The distinctive psychopathology of NMDAR-antibody encephalitis compared with primary psychoses: an international, multicentre, retrospective phenotypic analysis. *Lancet Psychiatry* 2026; **13:** 47–61.

Appendix

Supplementary Tables

Pages

1 Supplementary Table 1 – Para-clinical investigations – abnormal investigation result reported within episode
2 Supplementary Table 2 – Core clinical and para-clinical features inventory per episode reporting – denominator given if incomplete
3-4 Supplementary Table 3 – Psychopathologic feature inventory
5 Supplementary Table 4 – Past psychiatric history diagnoses per clinical group
6-7 Supplementary Table 5 – Psychopathology features in NMDAR-antibody encephalitis compared to pooled episodes of psychosis from early intervention cohort
8-9 Supplementary Table 6 – Diagnostic matrix used for classifier comparison
10 Supplementary Table 7 – Implementation of Possible Autoimmune Psychosis (Pollak et al 2020) criteria
11 Supplementary Table 8 – NMDAR-antibody encephalitis psychopathology score
12 Supplementary Table 9 – Predictive values of scoring methods as a function of selected prevalence values

Supplementary Figures

Pages

13-14 Supplementary Figure 1 – Graphical abstract
15-16 Supplementary Figure 2 – Journey through clinical services and treatment initiation in NMDAR-antibody encephalitis
17 Supplementary Figure 3 – Bar chart illustrating frequency of multiple factors in decision making to transfer patient from psychiatric care
18 Supplementary Figure 4 - Chord diagrams illustrating psychopathology feature co-occurrence
19-20 Supplementary Figure 5 - Analysis of NMDAR-antibody encephalitis and postpartum psychosis psychopathology time series data
21 Supplementary Figure 6 - Deriving a clinical score to differentiate NMDAR-antibody encephalitis from incident psychosis based on psychopathology data
22-23 Supplementary Figure 7 – Predictive values of scoring methods as a function of prevalence

Supplementary Methods

24-26

Supplementary Table 1 – Para-clinical investigations – abnormal investigation result reported within episode

	NMDAR-antibody encephalitis	Psychosis Unselected	Psychosis Selected	Post-partum psychosis
EEG	68	-	0/1	0/1*
CSF Protein	24	-	0/1	0/1*
CSF Pleocytosis	65	-	0/1	0/1*
MRI brain	44	-	-	0/1*

CSF = cerebrospinal fluid, EEG = electroencephalogram, MRI = magnetic resonance imaging,
*One patient was investigated by a neurologist due to seizure – sleep deprivation alongside psychotropic prescribing.

Supplementary Table 2 – Core clinical and para-clinical features inventory per episode reporting – denominator given if incomplete

**Question**	**Response**	**NMDAR-antibody-encephalitis** n=100 episodes	**Psychosis Unselected** n=83 episodes	**Psychosis Selected** n=52 episodes	**Post-partum psychosis** n=10 episodes
Centre, case, episode Code	Alphanumeric	100	83	52	10
Main clinical source<br>*Direct +/- Healthcare record*<br>*Healthcare record only*	Yes/No	 71<br>29	 N/A<br>83	 N/A<br>52	 10<br>N/A
Age at onset	Age	100	83	52	10
Biological sex	Female/Male	100	83	52	10
NMDAR-Ab-E specific associations	OT<br>Post-HSVE	25<br>2	N/A	N/A	N/A
Prior psychiatric history	Any<br>Specific diagnosis	25<br>25	43<br>43	17<br>17	5<br>5
Initial diagnosis	Neurologic or Psychiatric<br>Specific diagnosis	99/100<br>94/99	83<br>83	52<br>52	10<br>9/10
Core features*	Present or Not<br>If present, timing	900<br>468/472	747<br>Psychiatric as below	468<br>Psychiatric as below	90<br>Psychiatric as below
Psychiatric onset time available<br>/Number of illness episodes with psychiatric syndrome	Timing	91/92	67/83	47/52	8/10
Timing of presentation to at least one clinical service**	Timing	100	N/A	N/A	N/A
Initial management in psychiatric hospital, factors influencing transfer to general hospital***	At least one factor(s)	36	N/A	N/A	N/A
Symptomatic treatment****	Yes or No<br>If used, timing	500<br>225/256	N/A	N/A	N/A
Immunotherapy*****	Yes or No<br>If used, timing	500<br>159/224	N/A	N/A	N/A

*As per methods, nine core features at an overt neurologic level: Prodrome, Psychiatric, Cognitive, Seizure(s), Speech, Movement disorder, Autonomic instability, Central hypoventilation, Reduced conscious level
**At least one of General Practitioner, Emergency Department, Mental health, Internal medicine, Neurology
***Psychopathology, Treatment-resistance, NMS-like drug reaction, Seizure(s), Movement disorder, Autonomic instability, Reduced consciousness, View of carer, Other
****Benzodiazepine, Anti-seizure, Anti-psychotic, Sedating anti-histamine, Anti-depressant
*****Steroid, Plasma exchange, IVIG, Cyclophosphamide, Rituximab

Abbreviations used: CSF = cerebrospinal fluid, EEG = electroencephalogram, N/A = not applicable, NMS = neuroleptic malignant syndrome

Supplementary Table 3 – Psychopathologic feature inventory

Mental State Examination cluster (n=8)	Psychopathologic feature (n=58)
Behaviour: Agitated-violent	Agitation
	Aggression
	Violent
	Screaming
Behaviour: Disorganised-catatonic	Disorganised-bizarre
	Incongruent laughter-crying
	Talking to self
	Wandering
	Incoherent speech
	Repetitive speech
	Echolalia
	Mutism
	Stupor
	Blank expression
	Waxy flexibility
	Posturing
Mood	Free floating anxiety
	Panic
	Depressed mood
	Mood instability
	Irritable
	Elated mood
	Grandiosity
	Pressured speech
	Flight of ideas
	Distractible
	Goal-directed activity
	Impulsivity
	Disinhibition
	Hyper-sexuality
	Hyper-religiosity
	Mixed affect
Sleep	Insomnia
	Less need for sleep
	Hypersomnia
	Sleep-wake reversal
Appetite	Hypo-phagia
	Hyper-phagia
	Polydipsia
Thought	Delusional atmosphere
	Delusion (any)
	Paranoid delusion
	Grandiose delusion

	Other theme delusion
	Thought insertion
	Thought withdrawal
	Thought broadcast
	Passivity
Perception	Hallucinations (any)
	Auditory hallucinations only
	Visual hallucinations only
	Auditory and visual hallucinations
	3rd person auditory hallucinations
	Thought echo
	Running commentary
Suicidality	Self harm
	Suicidal thoughts
	Suicide attempt

Supplementary Table 4 – Past psychiatric history diagnoses per clinical group

Diagnosis	NMDAR-antibody encephalitis	Unselected psychosis	Selected psychosis	Post-partum psychosis
Total, n diagnoses	**29**	**51**	**17**	**5**
Common mental health condition				
Depression	8	18	11	2
Anxiety	6	11	3	0
Panic attacks	2	2	0	0
Post-partum depression	0	0	0	1
Sub-total, n diagnoses<br>% Total	*16*<br>***55***	*31*<br>***61***	*14*<br>***82***	*3*<br>***60***
Severe mental illness				
Schizophrenia	1	1	0	0
Bipolar disorder	5	0	2	0
Schizoaffective disorder	0	1	0	0
Delusional disorder	0	1	1	0
Post-partum psychosis	0	0	0	2
Sub-total, n diagnoses<br>% Total	*6*<br>***21***	*3*<br>***6***	*3*<br>***18***	*2*<br>***40***
Neurodevelopmental				
Autism spectrum disorder	2	4	1	0
Attention deficit hyperactivity disorder	3	4	0	0
Intellectual disability	0	1	0	0
Sub-total, n diagnoses<br>% Total	*5*<br>***17***	*9*<br>***18***	*0*<br>***0***	*0*<br>***0***
Personality				
Emotionally unstable personality disorder	0	4	0	0
Self harm	0	1	0	0
Conduct disorder	0	1	0	0
Sub-total, n diagnoses<br>% Total	*0*<br>***0***	*6*<br>***12***	*0*<br>***0***	*0*<br>***0***
Other				
Eating disorder	2	0	0	0
Obsessive-compulsive disorder	0	1	0	0
Seasonal affective disorder	0	1	0	0
Sub-total, n diagnoses<br>% Total	*2*<br>***7***	*2*<br>***4***	*0*<br>***0***	*0*<br>***0***

Percentages rounded to nearest integer.

Supplementary Table 5 – Psychopathology features in NMDAR-antibody encephalitis compared to pooled episodes of psychosis from early intervention cohort

Feature	OR	95%CI upper	95%CI lower	Unadjusted P	Rank (i)	BH Critical value	P < BH Critical value?
Screaming	6.09	13.85	2.73	0.0001	1	0.0009	Yes
Echolalia	Inf	Inf	4.37	0.0001	2	0.0017	Yes
Posturing	Inf	Inf	7.45	0.0001	3	0.0026	Yes
Stupor	31.83	334.70	5.44	0.0001	4	0.0034	Yes
Mutism	Inf	Inf	11.37	0.0001	5	0.0043	Yes
Blank expression	29.78	314.00	5.04	0.0001	6	0.0052	Yes
Repetitive speech	31.83	334.70	5.44	0.0001	7	0.0060	Yes
Wandering	7.66	18.49	3.04	0.0001	8	0.0069	Yes
Grandiosity	0.14	0.42	0.05	0.0001	9	0.0078	Yes
Less need for sleep	0.23	0.44	0.12	0.0001	10	0.0086	Yes
Paranoid delusion	0.16	0.28	0.09	0.0001	11	0.0095	Yes
Delusion (any)	0.12	0.22	0.07	0.0001	12	0.0103	Yes
Visual hallucinations only	12.28	33.39	4.38	0.0001	13	0.0112	Yes
Hypo-phagia	14.86	47.63	4.47	0.0001	14	0.0121	Yes
Grandiose delusion	0.20	0.48	0.08	0.0002	15	0.0129	Yes
Sleep-wake reversal	Inf	Inf	3.76	0.0003	16	0.0138	Yes
Delusional atmosphere	3.58	7.35	1.74	0.0003	17	0.0147	Yes
Flight of ideas	0.23	0.55	0.10	0.0008	18	0.0155	Yes
Free-floating anxiety	2.48	4.22	1.43	0.0013	19	0.0164	Yes
Waxy flexibility	Inf	Inf	2.60	0.0020	20	0.0172	Yes
Talking to self	3.37	7.29	1.54	0.0030	21	0.0181	Yes
Disorganised-bizarre	2.55	4.69	1.37	0.0032	22	0.0190	Yes
Auditory hallucinations only	0.39	0.72	0.21	0.0033	23	0.0198	Yes
Incongruent laughter-crying	3.04	6.61	1.43	0.0063	24	0.0207	Yes
Auditory and visual hallucinations	3.04	6.61	1.43	0.0063	25	0.0216	Yes
Incoherent speech	4.52	13.07	1.39	0.0080	26	0.0224	Yes
Panic	2.78	6.18	1.21	0.0146	27	0.0233	Yes
Thought broadcast	0.18	0.69	0.04	0.0161	28	0.0241	Yes
Suicidal thoughts	0.44	0.85	0.22	0.0165	29	0.0250	Yes
Mixed affect	4.94	17.03	1.39	0.0174	30	0.0259	Yes
Elated mood	0.39	0.87	0.18	0.0239	31	0.0267	Yes
Thought withdrawal	0.13	0.76	0.01	0.0270	32	0.0276	Yes
Insomnia	1.86	3.33	1.05	0.0385	33	0.0284	No
Thought insertion	0.21	0.87	0.05	0.0472	34	0.0293	No
Polydipsia	Inf	Inf	1.19	0.0744	35	0.0302	No
Pressure of speech	0.53	1.10	0.27	0.0885	36	0.0310	No
Goal-directed activity	0.32	1.04	0.09	0.1035	37	0.0319	No
Impulsivity	1.66	2.96	0.89	0.1211	38	0.0328	No
Hyper-religiosity	0.49	1.18	0.19	0.1394	39	0.0336	No
Disinhibition	1.65	3.16	0.87	0.1416	40	0.0345	No
Aggression	1.50	2.49	0.88	0.1424	41	0.0353	No
Hallucinations (any)	1.50	2.49	0.88	0.1451	42	0.0362	No

3rd person auditory hallucinations	0.35	1.19	0.10	0.1616	43	0.0371	No
Hyper-phagia	5.64	69.48	0.91	0.1654	44	0.0379	No
Thought echo	Inf	Inf	0.63	0.1779	45	0.0388	No
Hyper-sexuality	2.15	6.26	0.77	0.1814	46	0.0397	No
Passivity	0.43	1.39	0.15	0.1926	47	0.0405	No
Irritable	0.68	1.17	0.39	0.2156	48	0.0414	No
Suicide attempt	0.50	1.40	0.19	0.2233	49	0.0422	No
Hyper-somnia	1.87	7.55	0.49	0.4580	50	0.0431	No
Running commentary	0.45	3.06	0.03	0.6396	51	0.0440	No
Distractible	1.11	2.17	0.59	0.7408	52	0.0448	No
Violent	1.13	1.99	0.62	0.7658	53	0.0457	No
Other theme delusion	1.18	3.28	0.38	0.7804	54	0.0466	No
Depressed	0.90	1.54	0.54	0.7886	55	0.0474	No
Mood instability	1.11	1.89	0.65	0.7899	56	0.0483	No
Agitation	1.08	1.84	0.64	0.7920	57	0.0491	No
Self harm	0.86	2.09	0.36	0.8264	58	0.0500	No

Odds ratios (OR) and 95% confidence interval (CI) given to two decimal places, Unadjusted P values to four decimal places, and Benjamini-Hochberg (BH) critical value to three decimal places; ranked by P value in ascending order; where i (P-value rank), m (total number of tests) = 58, Q (false discovery rate) = 0.05, and critical value=(i/m)*Q. Inf=infinite.

Supplementary Table 6 – Diagnostic matrix used for classifier comparison

1 = inclusion criterion (permits diagnosis)
0·5 = supportive (common occurrence)
0 = no particular diagnostic relevance
-2 = exclusion criterion (rules out diagnosis)

**Disorder**	**Severe Depression**	**Mania**	**Psychotic depression**	**Psychotic mania**	**Schizophrenia**	**Cycloid psychosis**	**Atypical psychosis**	**Acute and transient psychotic disorder**	**Hebephrenia**	**Catatonia**
Reference	ICD11 6A70.3	ICD11 6A60.0	ICD11 6A70.4	ICD11 6A60.1	ICD11 6A20	Perris & Brockington 1981	Hinotsu et al 2022	ICD11 6A23	ICD10 F20.1	ICD11 6A4
Free floating anxiety	0·5	0·5	0·5	0·5	0	1	1	0	0	0
Panic attacks	0	0	0	0	0	0·5	0·5	0	0	0
Depressed mood	1	-2	1	-2	0	0	0	0	0·5	0
Mood instability/lability	0	1	0	1	0	1	1	0	1	0
Irritability	0·5	1	0·5	1	0	0·5	1	0	0·5	0
Elated mood	-2	1	-2	1	-2	0·5	0·5	-2	-2	0
Grandiosity	-2	1	-2	1	0	0	0	0	0	0
Pressure of speech	-2	1	-2	1	-2	0	0	0	0	0
Flight of ideas/racing thoughts	-2	1	-2	1	-2	0·5	0	0	0	0
Distractibility	0	1	0	1	0	0	0	0	0	0
Increased goal-directed activity	-2	1	-2	1	-2	-2	0	-2	-2	0
Impulsivity	0	1	0	1	0	1	0	0	0·5	0
Disinhibition	-2	1	-2	1	0	0	0	0	1	0
Hyper-sexuality	-2	1	-2	1	0	0	0	0	0·5	0
Hyper-religiosity	-2	1	-2	1	0	0	0	0	0·5	0
Subjectively less need for sleep	-2	1	-2	1	-2	0	0	-2	-2	0
Severe insomnia	1	0	1	0	0·5	0·5	0·5	0·5	0·5	0
Hypersomnia	0·5	0	0·5	0	0	0	0	0	0	0
Sleep-wake reversal	0	0	0	0	0	0	0	0	0	0
Disorganised/bizarre	0	0	0	0	1	0·5	0·5	0·5	1	0·5
Incongruent laughter/crying	0	0	0	0	0·5	0·5	0	0·5	1	0
Repetitive speech/verbigeration	0	0	0	0	0·5	0·5	0·5	0·5	0·5	1
Incoherent speech	-2	0·5	-2	0·5	1	0·5	0	1	1	1
Blank facial expression	0	0	0	0	0	0·5	0·5	0	0	1
Staring blankly	0	0	0	0	0	0·5	0·5	0	0	1

Mutism	0	0	0	0	0·5	0·5	1	0·5	0	1
Stupor	0	0	0	0	0·5	0·5	1	0·5	0	1
Catatonic motor	0	0	0	0	0·5	0·5	1	0·5	0	1
Echolalia	0	0	0	0	0·5	0·5	1	0·5	0	1
Delusional mood/atmosphere	-2	-2	0·5	0·5	1	1	1	1	1	0
Perplexity	-2	-2	0·5	0·5	1	1	1	1	1	0·5
Hallucinations	-2	-2	1	1	1	1	1	1	0·5	0
Non-auditory, non-visual hallucinations	-2	-2	1	1	1	1	1	1	0·5	0
Visual hallucinations only	-2	-2	0	0	0	0	0	0	0	0
Auditory hallucinations only	-2	-2	1	1	1	1	1	1	0·5	0
Both Auditory + visual hallucinations	-2	-2	0	0	0	0·5	1	0	0	0
Musical and/or noise hallucinations	-2	-2	0	0	0	0·5	0·5	0·5	0·5	0
Delusional perception	-2	-2	0	0	1	0·5	0·5	0·5	0·5	0
Auditory hallucinations in 3rd person	-2	-2	0	0	1	0·5	0·5	0·5	0·5	0
Auditory hallucination of running commentary	-2	-2	0	0	1	0·5	0·5	0·5	0·5	0
Auditory hallucination of thought echo	-2	-2	0	0	1	0·5	0·5	0·5	0·5	0
Somatic hallucinations	-2	-2	0·5	0·5	0·5	0·5	0·5	0·5	0·5	0
Delusion/overvalued ideas (any)	-2	-2	1	1	1	1	1	1	0·5	0
Paranoid/persecutory	-2	-2	0	0·5	1	1	1	1	0·5	0
Grandiose	-2	-2	0	1	0	0	0·5	0	0·5	0
Other theme	-2	-2	1	0·5	0	0·5	0·5	0	0	0
Thought insertion	-2	-2	-2	0·5	1	0·5	0·5	0·5	0·5	0
Thought withdrawal	-2	-2	-2	0·5	1	0·5	0·5	0·5	0·5	0
Thought broadcast	-2	-2	-2	0·5	1	0·5	0·5	0·5	0·5	0
Passivity	-2	-2	0	0·5	1	0·5	0·5	0·5	0·5	0
Agitation	0·5	0·5	0·5	0·5	0·5	0·5	0·5	0·5	0·5	0·5
Aggression	0	0	0	0	0	0	0	0	0	0
Violent	0	0	0	0	0	0	0	0	0	0
Suicidal thoughts	1	0·5	1	0·5	0·5	0	0	0·5	0	0
Suicide attempt	1	0·5	1	0·5	0·5	0	0	0·5	0	0
Hypo-phagia	0·5	0	0·5	0	0	0	0	0	0	0
Hyper-phagia	0·5	0	0·5	0	0	0	0	0	0	0
Polydipsia	0	0	0	0	0·5	0	0	0·5	0	0

Supplementary Table 7 – Implementation of Possible Autoimmune Psychosis (Pollak et al 2020) criteria

Category	Detail	Modification from Pollak et al?
**1st step**		
Onset	Within 3 months	No
Psychiatric	Psychotic symptoms	Yes<br>Broadened to any psychiatric feature
**2nd step ≥1 feature**		
Neurologic	Reduced consciousness	No
Neurologic	Severe cognitive dysfunction	No
Neurologic	Seizure(s)*	No
Neurologic	Autonomic instability	No
Neurologic	Movement disorder	Yes<br>Catatonia treated separately from movement disorder
Psychiatric	Catatonia	As above
Psychiatric	Neuroleptic sensitivity	No
Other	Current or recent tumour diagnosis	Yes<br>Restricted to ovarian teratoma

*Not explained by a previously known seizure disorder

We implemented the Possible Autoimmune Psychosis (Pollak) criteria as above, requiring firstly rapid psychiatric onset (<91 days), and secondly one or more of the clinical features listed.

Supplementary Table 8 – NMDAR-antibody encephalitis psychopathology score

**Feature**	**Present**
Rapid psychiatric symptom onset and progression (<six days)	+2
Female sex	+1
Sleep-wake reversal	+1
Stupor	+1
Screaming	+1
Repetitive speech	+1
Posturing	+1
Incoherent speech	+1
Mutism	+1
Delusions (any)	-1
Paranoid delusion	-1
Elated mood	-1
Flight of ideas	-1

Supplementary Table 9 – Predictive values of scoring methods as a function of selected prevalence values

Test	Prevalence	Baseline PPV (%)	PPV (%)	PPV Gain (%)	Baseline NPV (%)	NPV (%)	NPV Gain (%)
AP - community	1%	1.0	49.75	+48.75	99.0	99.98	+0.98
AP - pooled		1.0	9.91	+8.91	99.0	99.98	+0.98
Psychopathology – test set		1.0	12.39	+11.39	99.0	99.98	+0.98
AP - community	5%	5.0	83.76	+78.76	95.0	99.89	+4.89
AP - pooled		5.0	36.43	+31.43	95.0	99.88	+4.88
Psychopathology – test set		5.0	42.42	+37.42	95.0	99.89	+4.89
AP - community	25%	25.0	97.03	+72.03	75.0	99.33	+24.33
AP - pooled		25.0	88.29	+63.29	75.0	97.44	+22.44
Psychopathology – test set		25.0	90.74	+65.74	75.0	97.54	+22.54
AP - community	50%	50.0	98.99	+48.99	50.0	98.02	+48.02
AP - pooled		50.0	91.59	+41.59	50.0	97.85	+47.85
Psychopathology – test set		50.0	93.33	+43.33	50.0	97.89	+47.89

Abbreviations used: AP=autoimmune psychosis, NPV=negative predictive value, PPV=positive predictive value

Supplementary Figure 1 – Graphical abstract

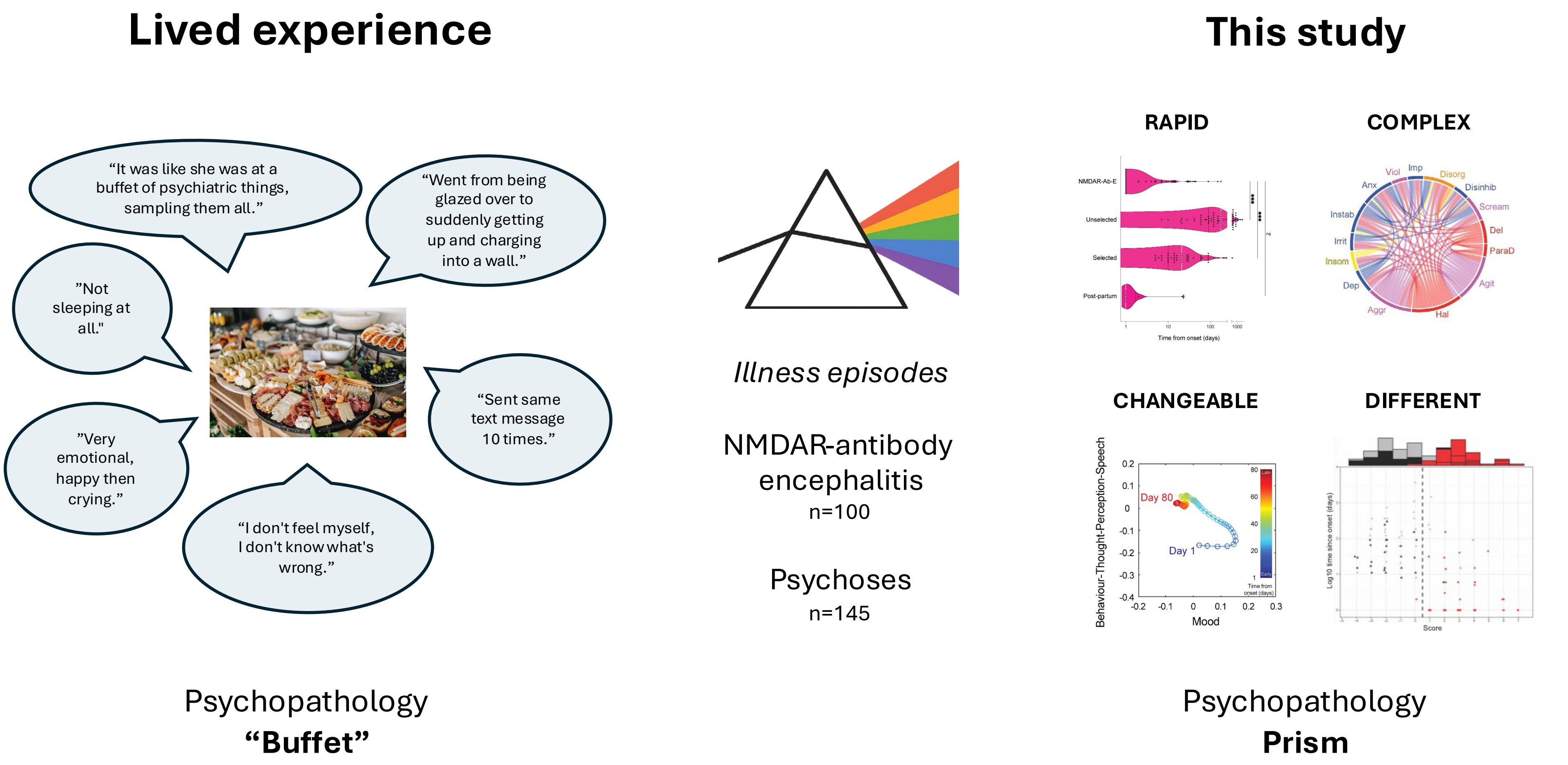

The origins, implementation, and key findings of the study are summarised as a graphical abstract. Left – Key lived experience comments are noted giving an insight into the nature of the psychiatric onset of NMDAR-antibody encephalitis. The picture of a buffet derived from one account conveys the variety of features brought together. Right – The current study sought to use psychopathology as a prism to make this experience granular to then re-construct on a quantitative basis in order to more formally test the intuitive impression. Four key sub-figures are presented from top to bottom: a rapid rate of onset, a complex overlap of features, features changeable over time, and overall sufficient differences to differentiate by a reductive score-based approach.

Buffet image reproduced unmodified from Wikimedia commons, Ivan Radic (CC-BY-2.0):
https://commons.wikimedia.org/wiki/File:Buffet_table_with_smoked_meat_and_cheese_plate,_appetizers_and_other_finger_food_-_Flickr_-_Ivan_Radic.jpg

Supplementary Figure 2 – Journey through clinical services and treatment initiation in NMDAR-antibody encephalitis

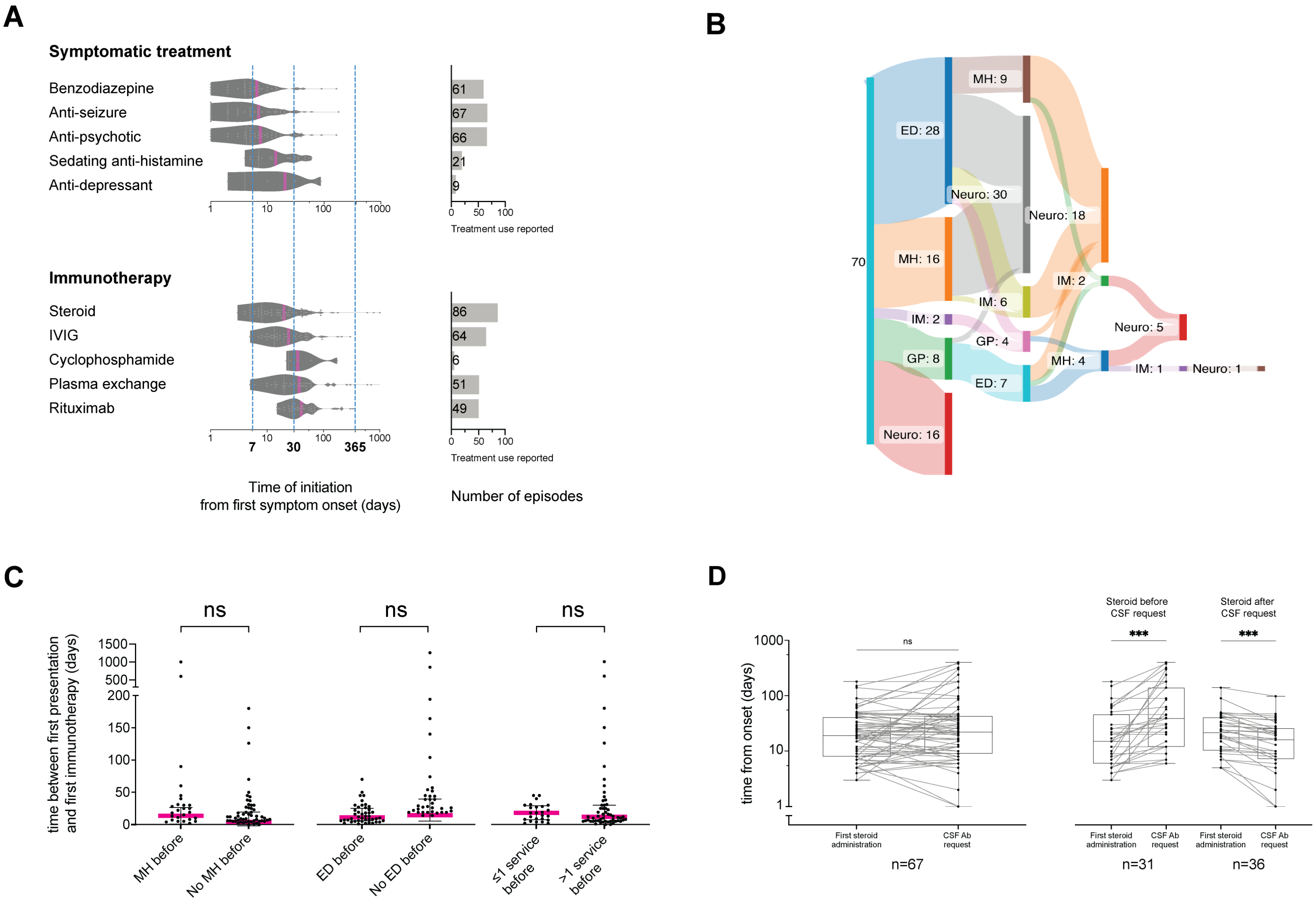

A – Violin plots summarise the time course of clinical presentation and treatment from episode onset on a logarithmic time axis (left column). The broad categories are ranked top to bottom by overall time from onset of the components within, which are in turn ranked top to bottom by earliest to latest median time from onset. Alongside (right column) horizontal bar charts summarise positively reported instances. Dots = individual illness episode, vertical pink bar = median, vertical grey bars = upper and lower limits of interquartile range; on the X axis 7, 30, and 365 days are emphasised in bold with a vertical dashed blue bar to demarcate one week, month, and year, respectively.

B – A Sankey plot summarises on patient transition between clinical services, where sequence available.

Abbreviations used: Ab=antibody, CSF=cerebrospinal fluid, ED=emergency department, GP=general practitioner, IM=internal medicine, MH=mental health service, MRI=magnetic resonance imaging, Neuro=neurology service, ns=not significant, PLEX=plasma exchange, Rep.=investigation reported, Req.=investigation requested, RTX=rituximab

C – Scatter plots summarise time between first presentation and first immunotherapy in three comparisons of the journey prior to treatment: seeing a mental health professional, attending the emergency department, or greater than one clinical service.

D – Box and whisker plots of timing of first steroid administration and CSF NMDAR-antibody request (median=horizontal line, box=interquartile range, whiskers=minimum-maximum) with individual episodes indicated by black circles linked grey lines to show the relationship between treatment and test request. Left – overall data and Right – split into episodes where steroid administered before or on same day as test or after.

All comparisons of median times made using a Two-tailed Wilcoxon matched-pairs signed rank test.

ns=not significant, ***P<0·001

Supplementary Figure 3 – Bar chart illustrating frequency of multiple factors in decision making to transfer patient from psychiatric care

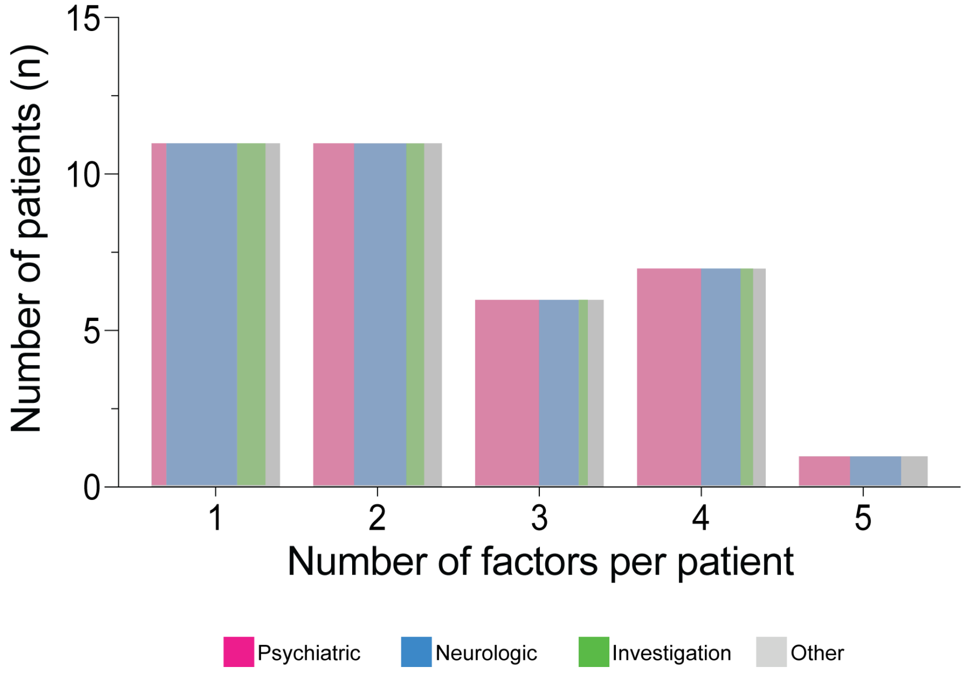

The frequency of the number of factors identified per illness episode are plotted on a vertical bar chart inset with category scaled proportionately. The broad category of the factor is colour coded as pink for psychiatric, blue for neurologic, green for investigation finding, and grey for others.

Supplementary Figure 4 - Chord diagrams illustrating psychopathology feature co-occurrence

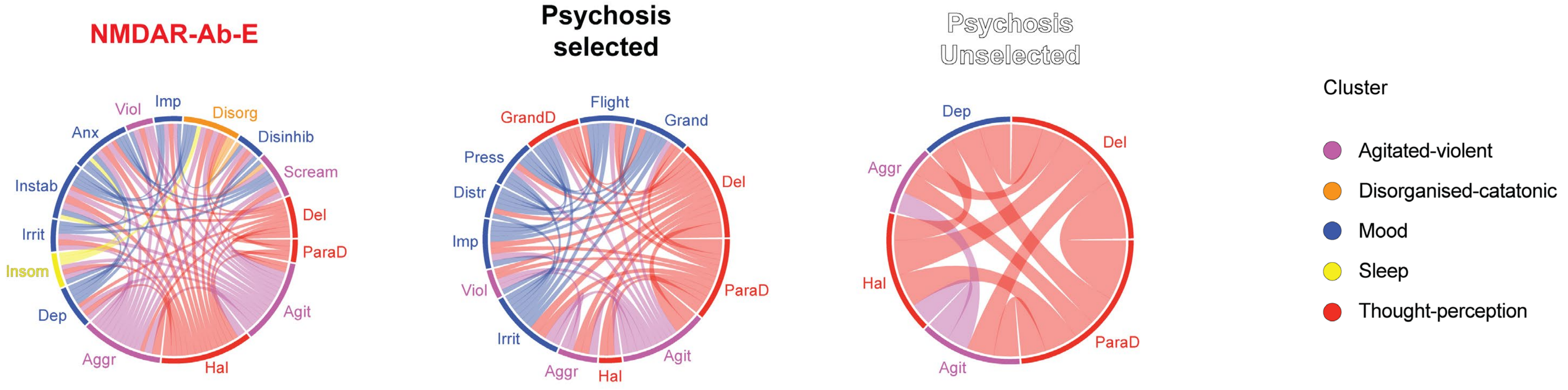

Within episode co-occurrent mental state feature pairs are summarised in chord diagrams thresholded to include feature pairs with more co-occurrences than 30% of the cohort size based upon individual links. The surviving features are labelled and colour-coded by higher level feature clusters as per the legend. Each ribbon represents the occurrence of at least one pair.

Abbreviations used: Aggr = Aggression, Agit = Agitation, Anx = Free floating anxiety, Del = Delusion (any), Dep = Depressed mood, Disinhib= Disinhibition, Disorg = Disorganised-bizarre, Distr = Distractable, Flight = Flight of ideas, Grand = Grandiosity, GrandD = Grandiose delusion, Hal = Hallucinations (any), Inf = infinity, Imp = Impulsivity, Insom = Insomnia, Instab = Mood instability, Irrit = Irritable, ParaD = Paranoid delusion, Press = Pressured speech, Scream = Screaming, tSNE = t-distributed stochastic neighbour embedding, Viol = Violent.

Supplementary Figure 5 - Analysis of NMDAR-antibody encephalitis and postpartum psychosis psychopathology time series data

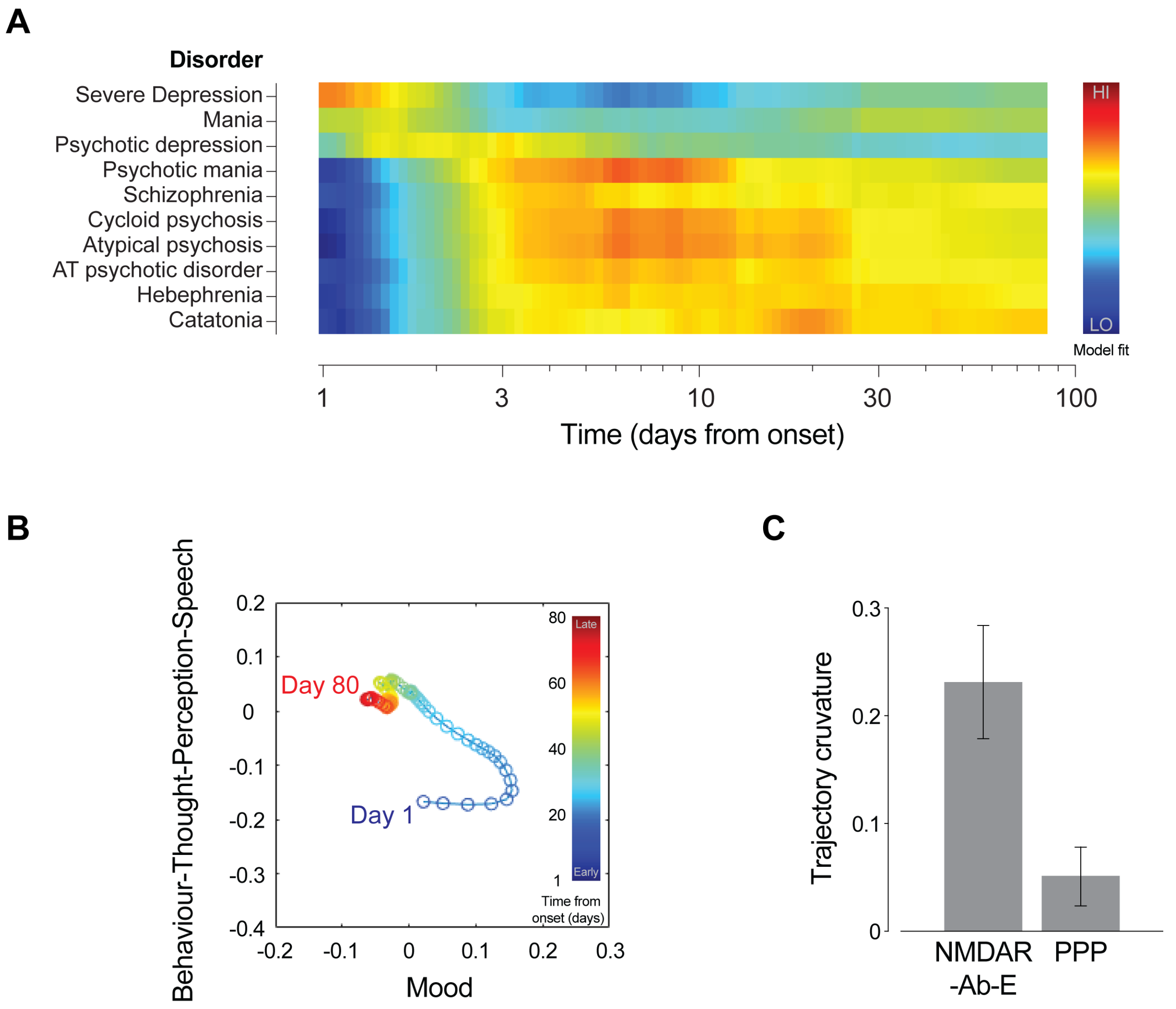

A – Time-windowed NMDAR-Ab-E mental state features were compared with 10 diagnostic categories using non-competitive distance-normalised weightings. The level of model fit is colour-coded and plotted for each diagnostic category on a logarithmic time axis (red=high fit, blue=low fit).

B – Mental state features for NMDAR-Ab-E episodes are reduced into two psychopathologic dimensions (x = mood and y = remainder) and an average plotted by onset time (Blue=early, Red=late).

C – The mean area enclosed by the curve of the two principal components is summarised on a bar plot for NMDAR-Ab-E as compared to postpartum psychosis (error bars=standard deviation).

Abbreviation used: NMDAR-Ab-E = NMDAR-antibody encephalitis, PPP = postpartum psychosis

Supplementary Figure 6 - Deriving a clinical score to differentiate NMDAR-antibody encephalitis from incident psychosis based on psychopathology data

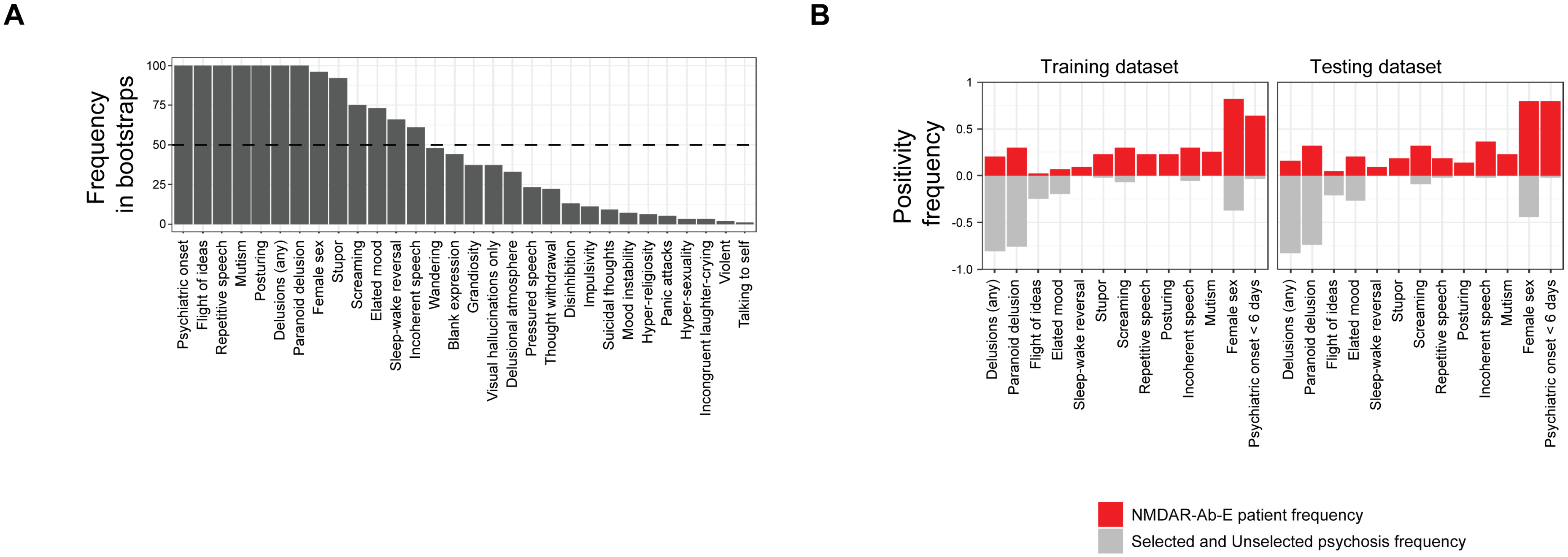

A – Frequency of features that discriminated between FEP and NMDAR-Ab-E after running a random-forest selection tool 100 times, randomly excluding 20% of the data in each round. The dashed horizontal line separates features that were identified in more than 50% of the runs.

B – Frequency of positivity for each of the thirteen items in the constructed psychopathology score. Red bars indicate frequency for NMDA-R-Ab-E patients, whereas grey bars indicate frequency for psychosis patients (selected and unselected together). Based on these frequencies, the first four features give negative points, whereas the remaining features give positive points in the score. Train and test sets are shown separately, indicating similar distributions for all features in both data subsets.

Supplementary Figure 7 – Predictive values of scoring methods as a function of prevalence

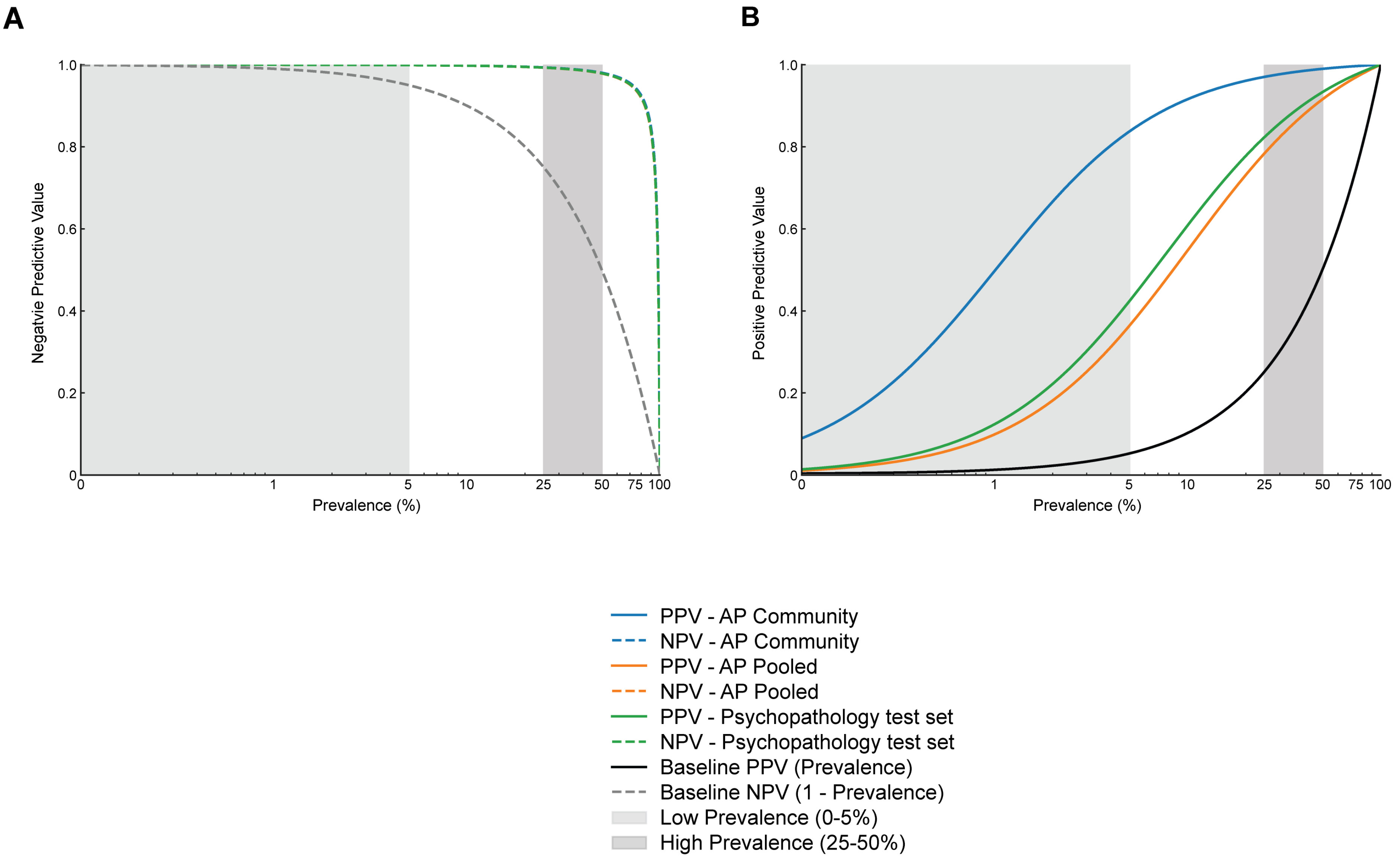

A, negative and B, positive predictive values (Y-axis, Linear scale) based on test accuracy (sensitivity and specificity) for three different approaches (Possible autoimmune psychosis vs community [blue] or pooled comparators [orange], and the test proportion of the psychopathology-based score vs pooled comparators [green]) as a function of prevalence (X-axis, Log scale). Positive predictive values are solid lines and negative predictive values are dashed lines. Regions of low and high prevalence are indicated by grey shading (Light grey=0-5%, Dark grey=25-50%).

Formulae used:
Negative Predictive Value = Specificity X (1-Prevalence) / (Specificity X (1-Prevalence)) + (1- Sensitivity) X Prevalence
Positive Predictive Value = Sensitivity X Prevalence / (Sensitivity X Prevalence) + (1-Specificity) X (1-Prevalence)
To plot predictive value entirely as a function of prevalence, sensitivity and specificity assumed=0.5.

Abbreviations used: AP=autoimmune psychosis, NPV=negative predictive value, PPV=positive predictive value

Supplementary methods

tSNE plot

To visualise how the pattern of features distinguished between individuals, we used an unsupervised dimensionality reduction method, t-stochastic neighbour embedding (tSNE, MATLAB Stats toolbox). This method visualises samples in the ambient 58-dimensional mental state feature space as a two-dimensional planar representation, such that with high probability, the distance between similar and different samples in the visualisation respect the same similar/different relationships in the original, ambient feature space.

Diagnostic classification matrix

We selected 10 psychiatric diagnoses spanning a spectrum of mood, psychotic and mixed disorders encompassing working diagnoses initially given to NMDAR-Ab-E patients and comparisons made in the published literature (Supplementary Table 6). This included ICD-11 diagnoses (ICD-10 for hebephrenia) and for atypical conditions we used key publications (Supplementary References). For each diagnosis, we adopted a weighting for each of the 58 features and how they could contribute to that diagnosis. These weights were coded as "should be present", "could be present", "must be absent", or "neither for nor against" (+1, +0.5, -2, 0, respectively). We optimised this with stereotyped exemplar cases and then proceeded to analyse our dataset.

Non-competitive distance-normalised weightings

We developed a method of projecting the symptom vector onto operationalised psychiatric diagnoses. The inner product of the weights with the features resulted in an index of how well each operationalised diagnosis fits the pattern of features. This score would be positive if the pattern of features matched the diagnosis, and negative if it would not. To examine the way in which operationalised diagnoses unfolded over time in NMDAR-Ab-E, we generated a feature vector for each day, from day 1 until day 80. The feature was considered to be +1 after the start time and before the end time, and 0 at other times. Where a feature had a start date but no recorded end date, it was considered to be present until day 80. Features were all 0 on day zero. The feature time series were smoothed using a gaussian kernel of standard deviation 2 days, before calculating the corresponding operationalised diagnosis indices. This enabled a continuous estimate of the relative likelihood of each diagnosis. The diagnoses were then normalised by z-scoring over time then dividing by the diagnosis vector length, so that the 10-dimensional diagnosis index had length 1. This meant that on any day, the index reflected the proportion of the total symptom intensity that was accounted for by each diagnosis. For each patient, this produced a temporal profile of the alignment of each diagnosis with the features present. The mean of these profiles indicates the average progression from one operationalised diagnosis to another over time.

Symptom space

To visualise the progression from mood to other dimensions over time, each feature was assigned into one of two categories: Mood / Affect, vs Thought / Behaviour / Perception / Speech. For each category, the total of each symptom present was calculated, and normalised by subtracting the mean over time. For each feature group, this produced a time-

course of how much this category accounted for more or less the overall presentation. This generated a 2-dimensional trajectory for each patient over time, indicting their balance of these two types of symptom over time.

Decision rule classifier

We selected all patients from the confirmed NMDAR-Ab-E cases, post-partum psychosis cases, first-episode psychosis consecutive cases and the first-episode acutely unwell cases. Patients where the timing of emergence of psychiatric behaviours variable was missing were excluded because by definition, these patients would not be considered to be in need of psychiatric consultation and assessment (a total of n=215 episodes were available comprising n=91 NMDAR-Ab-E, and n=124 primary psychosis). We operationalised the clinical features in the possible autoimmune psychosis criteria by matching them as closely as possible to the variates available in our dataset (Suppl. Table 7). We then tested decision rules that enabled classifying illness episodes as being probable for NMDAR-Ab-E or not in the context of a simulated community psychiatry caseload (unselected psychosis only, n=67) or higher acuity setting (unselected, selected, and postpartum psychosis, n=124).

Bayesian analyses of common criteria features

We sought to estimate the magnitude and direction of model coefficients (betas) of the probability of a given illness episode being NMDAR-Ab-E or not in an estimated general linear model (GLM). We included as independent variables the previous autoimmune psychosis features and also rapid onset of reduced consciousness and cognitive dysfunction, step 1 in the consensus possible autoimmune encephalitis approach.[28] We used Bayesian estimation because this delivers a posterior distribution for each parameter that can be examined to determine the most likely direction and magnitude for each predictor as well as exposing the uncertainty on each parameter estimate, e.g. the dispersion or concentration of the parameter's posterior distribution. Examining the posterior distributions of the parameters therefore provides more information about the stability of each clinical feature predictor to be useful in subsequent decision rule.

We used the *brms* package in R, wrapping the Stan/RStan library and followed recommendations for weakly-informative priors on the parameters in a logistic regression.[34] The prior used for all 11 included clinical feature parameters was a Student t-distribution with 7 degrees of freedom, location = 0 and scale = 2 for thinner tails of the distribution around a mean of 0. Posterior distributions for each parameter were visually inspected using ridge plots from the *bayesplot* package with median values and 80% probability mass intervals around the median.

Random-forest and score construction

Cases with complete data of 55 psychiatric variables, including time since onset, and biological sex were included (psychosis unselected=67, psychosis selected=47, and NMDAR-Ab-E=88). All the community psychosis patients were grouped for the downstream analyses. This dataset was then divided into training and test halves, with postpartum psychosis cases left for testing. To identify features that robustly contribute to separation of the NMDAR-Ab-E from the psychosis group, a bootstrapped, random forest-based feature selection method from the *Boruta* R package was used on 80% of the training data. This procedure was repeated 100 times, randomly excluding 20% of the data in each round. Features that were identified in more than 50% of the runs were retained. For the time variable, the optimal number of days since onset as well as the

optimal weight were identified in a combinatorial scheme, testing a range of cutoffs for time from 1 to 90 days and a weight for the time variable from 1 to 5, where the readout was the highest balanced accuracy for the training data. After the selection of the features and the weight for the time since onset, threshold for separation of the groups was identified that again optimised the balanced accuracy for the training data.

References

Diagnostic classification

Al-Diwani A, Handel A, Townsend L, et al. The psychopathology of NMDAR-antibody encephalitis in adults: a systematic review and phenotypic analysis of individual patient data. The Lancet Psychiatry. 2019;6(3):235-46.

Perris C BI. Cycloid psychoses and their relation to the major psychoses. in Biological Psychiatry Edited by Perris C, Struwe D, Janson B Amsterdam, Elsevier. 1981:pp 447–50.

Gine Serven E, Boix Quintana E, Martinez Ramirez M, et al. Cycloid psychosis as a psychiatric expression of anti-NMDAR encephalitis. A systematic review of case reports accomplished with the authors' cooperation. Brain Behav. 2021;11(2):e01980.

Heron J, McGuinness M, Blackmore ER, Craddock N, Jones I. Early postpartum symptoms in puerperal psychosis. BJOG. 2008;115(3):348-53.

Hinotsu K, Miyaji C, Yada Y, et al. The validity of atypical psychosis diagnostic criteria to detect anti-NMDA receptor encephalitis with psychiatric symptoms. Schizophr Res. 2022;248:292-9.

Perris C BI. Cycloid psychoses and their relation to the major psychoses. in Biological Psychiatry Edited by Perris C, Struwe D, Janson B Amsterdam, Elsevier. 1981:pp 447–50.

R Packages

Bayesplot	Gabry J, Mahr T (2025). "bayesplot: Plotting for Bayesian Models." R package version 1.14.0 https://github.com/stan-dev/stan/wiki/prior-choice-recommendations. Accessed 15.9.25.
Boruta	Witold R. Rudnicki M (2010). "Feature Selection with the Boruta Package." Journal of Statistical Software, 36(11), 1–13.
Brms	Bürkner P (2021). "Bayesian Item Response Modeling in R with brms and Stan." Journal of Statistical Software, 100(5), 1–54.
